# Neuroglobin overexpression in cerebellar neurons of *Harlequin* mice improves mitochondrial homeostasis and reduces ataxic behavior

**DOI:** 10.1016/j.ymthe.2024.05.030

**Published:** 2024-05-24

**Authors:** Hélène Cwerman-Thibault, Vassilissa Malko-Baverel, Gwendoline Le Guilloux, Edward Ratcliffe, Djmila Mouri, Isabel Torres-Cuevas, Ivan Millán, Bruno Saubaméa, Virginie Mignon, Odile Boespflug-Tanguy, Pierre Gressens, Marisol Corral-Debrinski

**Affiliations:** 1 Université Paris Cité, Inserm, Maladies neurodéveloppementales et neurovasculaires, F-75019 Paris, France; 2 Neonatal Research Group, Health Research Institute La Fe, 46026 Valencia, Spain; 3 Laboratory of Comparative Neurobiology, Cavanilles Institute of Biodiversity and Evolutionary Biology, University of Valencia, Valencia, Spain; 4 Université Paris Cité, Platform of Cellular and Molecular Imaging (PICMO), US25 Inserm, UAR3612 CNRS, 75006 Paris, France; 5 Université Paris Cité, Optimisation Thérapeutique en Neuropsychopharmacologie, UMR-S 1144 Inserm, 75006 Paris, France; 6 Service de Neurologie et Maladies métaboliques, CHU Paris - Hôpital Robert Debré, F-75019 Paris, France

**Keywords:** neuroglobin, mitochondria, Purkinje cells, gene therapy, *Harlequin* mice, respiratory chain, AAV2/9 vectors, Apoptosis-inducing factor

## Abstract

Neuroglobin, a member of the globin superfamily, is abundant in the brain, retina, and cerebellum of mammals and localizes to mitochondria. The protein exhibits neuroprotective capacities by participating in electron transfer, oxygen supply, and protecting against oxidative stress. Our objective was to determine whether neuroglobin overexpression can be used to treat neurological disorders. We chose *Harlequin* mice, which harbor a retroviral insertion in the first intron of the *apoptosis-inducing factor* gene resulting in the depletion of the corresponding protein essential for mitochondrial biogenesis. Consequently, *Harlequin* mice display degeneration of the cerebellum and suffer from progressive blindness and ataxia. Cerebellar ataxia begins in *Harlequin* mice at the age of 4 months and is characterized by neuronal cell disappearance, bioenergetics failure, and motor and cognitive impairments, which aggravated with aging. Mice aged 2 months received adeno-associated viral vectors harboring the coding sequence of neuroglobin or *apoptosis-inducing factor* in both cerebellar hemispheres. Six months later, *Harlequin* mice exhibited substantial improvements in motor and cognitive skills; probably linked to the preservation of respiratory chain function, Purkinje cell numbers and connectivity. Thus, without sharing functional properties with apoptosis-inducing factor, neuroglobin was efficient in reducing ataxia in *Harlequin* mice.

## Graphical abstract

**Figure:**
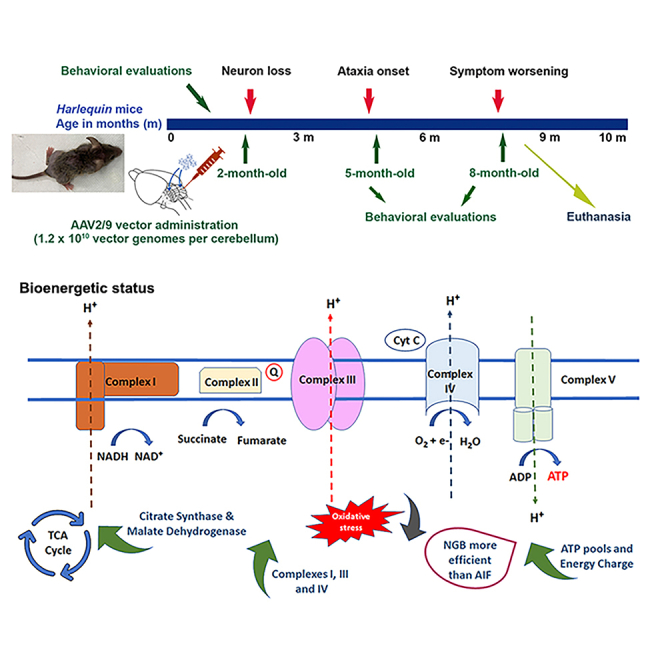

## Teaser

Progressive cerebellar ataxia begins in *Harlequin* mice at the age of 4 months, characterized by Purkinje cell disappearance, bioenergetics failure, oxidative stress, and motor and cognitive impairments. Substantial improvements of all these parameters were demonstrated in mice that received in their cerebella AAV2/9 vectors harboring the coding sequence of neuroglobin.

## Introduction

Mitochondria contain approximately 1,300 proteins encoded by nuclear and mitochondrial genomes.^1^^,^^2^ They perform essential functions in addition to the synthesis of adenosine triphosphate (ATP) via oxidative phosphorylation (OXPHOS), including calcium (Ca^2+^) buffering, biogenesis of lipids and iron-sulfur clusters, regulation of reactive oxygen species (ROS), hormone biosynthesis, and apoptosis.^3^

It is estimated that the adult human brain represents the largest source of energy consumption, approximately 20% of the body’s oxygen metabolism. The energetic cost of brain activities must be efficiently covered by mitochondrial respiration, to sustain and regulate neuronal activity and synaptic plasticity.^4^^,^^5^ Accordingly, small alterations in metabolic processes may severely affect neuronal integrity and increase their vulnerability. This is why a growing number of neurological diseases are associated with mitochondrial dysfunction.^6^^,^^7^

Mutations in genes encoding mitochondrial proteins lead to primary mitochondrial disorders, which represent one of the most common and debilitating inherited metabolic diseases often resulting in early mortality.^8^ To date, more than 350 genes have been causally linked to mitochondrial diseases.^9^ Patients with mitochondrial diseases suffer from multi-systemic symptoms, especially affecting organs with high energetic demands such as the brain, muscle, heart, retina, optic nerve, and liver.^10^

Not only OXPHOS dysfunction but also imbalanced mitochondrial dynamics,^11^ impaired mitochondrial lipid homeostasis,^12^ and altered redox status^13^ contribute to pathophysiology of mitochondrial diseases. In the last decades, a better understanding of the pathogenesis has been achieved leading to upgraded diagnoses and the implementation of suitable therapies.^14^^,^^15^ There is no cure or approved therapy currently available for mitochondrial diseases, certainly due to the complexity of phenotypes associated with mitochondrial dysfunction.^16^ Nevertheless, few symptomatic treatments have been proven by clinical trials such as combination of vitamins, cofactors, nutrients, and antioxidants, which may alleviate symptoms and limit disease progression.^17^^,^^18^^,^^19^ Besides, organ transplantation is used in the few cases in which patients suffer from an isolated organ failure.^20^

Accordingly, efforts are ongoing to lastingly preserve mitochondrial function in patients through innovative approaches including pharmacological treatments to enhance respiratory chain activity, to stimulate mitochondria biogenesis, or to preserve mitochondrial fusion/fission processes as well as gene therapy to correct the causing-gene mutation.^21^^,^^22^ Among them, gene therapy appears promising to treat devastating conditions due to neuronal cell death as recently shown for spinal muscular atrophy and Leber hereditary optic neuropathy.^23^^,^^24^

Neuroglobin (NGB) (encoded by the *Ngb* gene) was identified in 2000 as a member of the globin superfamily highly conserved throughout evolution.^25^ The protein of 151 amino acids is abundant in the brain, being present in both neurons^26^ and astrocytes.^27^ The neuroprotective role of neuroglobin has been largely demonstrated *in vitro* and *in vivo*.^28^^,^^29^ It is now well recognized that the majority of the protein localizes to the mitochondria, as we described in rodent retinas, in which we showed that NGB is required for respiratory chain function.^30^^,^^31^^,^^32^

As a recipient of gene therapy, we study *Harlequin* (*Hq*) mice that develop ataxia as they age^33^ in order to prevent cerebellar degeneration by *Ngb* overexpression.

*Harlequin* mice harbor a 9-kb insertion of the ecotropic leukemia provirus in the first intron of the X-linked apoptosis-inducing factor gene (*Aifm1*) resulting in an 80%–90% reduction of the corresponding protein (apoptosis-inducing factor [AIF]) abundance in all the tissues.^33^ *Harlequin* mice exhibit progressive degeneration of the retina, optic nerve, cerebellum, and cortical regions leading to blindness and ataxia, which begin in 4-month-old *Hq* mice.^33^^,^^34^^,^^35^

AIF is an FAD-dependent NADH oxidoreductase that localizes to the mitochondrial intermembrane space, with its N terminus anchored to the inner membrane. The protein interacts with MIA40/CHCHD4, the central component of a redox-sensitive mitochondrial intermembrane space import machinery.^36^^,^^37^ AIF regulates mitochondrial OXPHOS and energy homeostasis by assisting biogenesis and/or stabilization of respiratory chain complexes.^38^

We demonstrated severe alterations of mitochondrial morphology and function in cerebellar neurons of *Hq* mice aged 2 months, which aggravated with age and led to the diminution of motor and cognitive capabilities.^39^

Therefore, 2-month-old *Hq* mice were subjected to stereotactic surgery and each cerebellar hemisphere received adeno-associated viral vectors, serotype 9 (AAV2/9) harboring *Ngb* or *Aifm1* coding sequence.

Morphological evaluations indicated that cerebellum from *Hq* mice subjected to gene therapy displayed more Purkinje cells with a better preserved dendritic arborization in the molecular layer and increased axonal projections through the deep nuclei.

Gene therapy resulted in a significant improvement of cerebellar bioenergetic status (respiratory chain/tricarboxylic acid [TCA] cycle enzymatic activities or nucleotide pools) largely compromised in *Hq* mice treated with the AAV2/9-*GFP* vector, suggesting that gene therapy preserved mitochondrial homeostasis. Moreover, overexpression of *Ngb* is far more effective in counteracting the oxidative stress revealed in cerebella from these mice than that of *Aifm1*.

Importantly, both motor and cognitive skills were significantly improved in *Hq* mice subjected to gene therapy regardless of which vector was used.

Altogether, NGB appears a promising tool for designing clinical studies aiming to improve life conditions of patients suffering from an array of neurological diseases.

## Results

### Gene therapy diminished cerebellar atrophy in *Hq* mice

**Figure 1:**
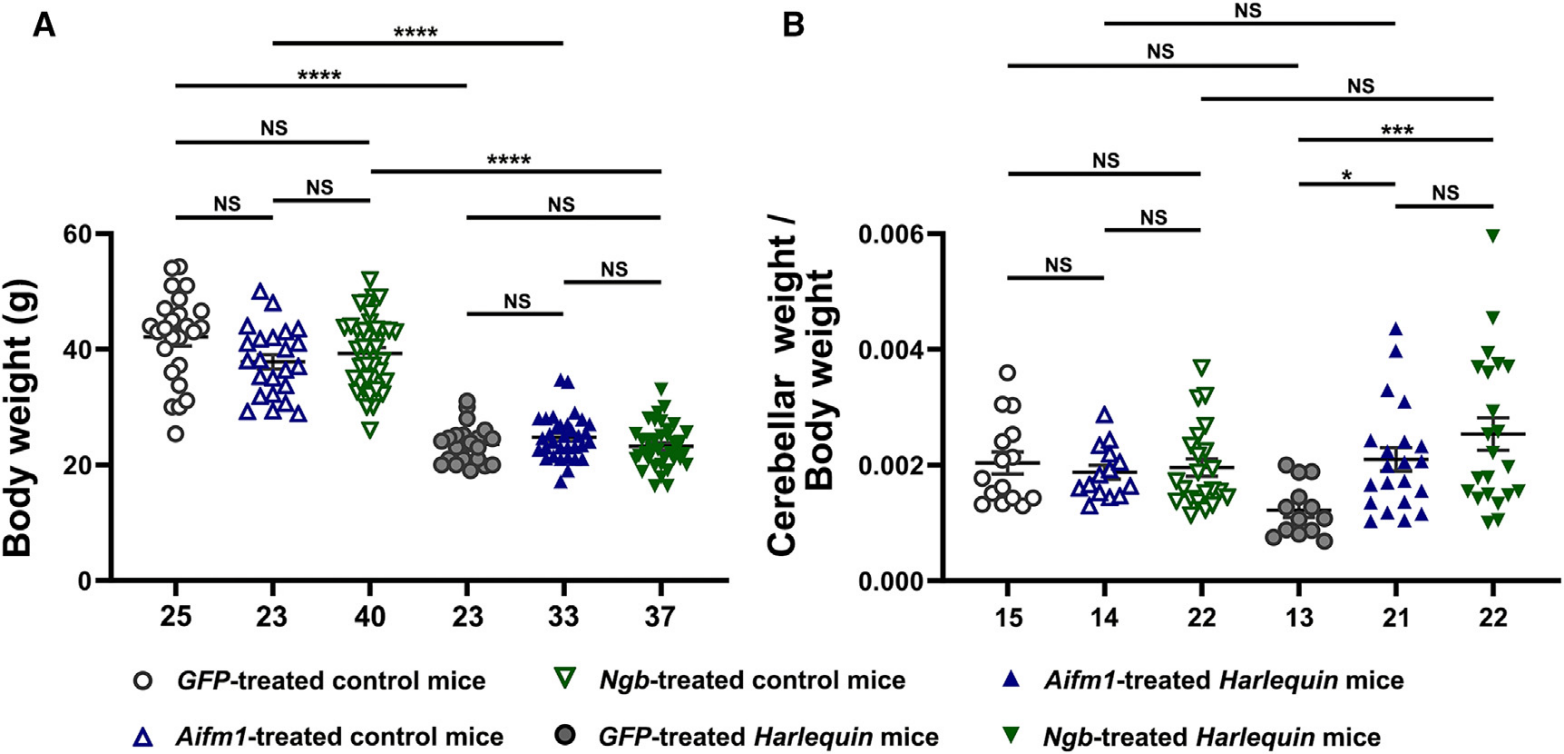
Body and cerebellar weights of *Harlequin* mice subjected to gene therapy Comparison of body and cerebellar weights in control and *Hq* mice subjected to gene therapy with AAV2/9-*GFP*, AAV2/9-*Aifm1*, or AAV2/9-*Ngb* vector, at the age of 2 months and euthanized 6 months later. (A) Body weights follow a normal distribution; thus, the two-way ANOVA test was used. (B) Ratios of cerebellar weights to body weights were calculated to compare cerebellar weights in mice subjected to gene therapy. Data follow a Gaussian distribution, so the two-way ANOVA test was applied.The number of mice for each group evaluated appears below each column of the histograms. To compare the different groups, the GraphPad Prism 10.2 software was used. Adjusted *p* values in each histogram are as follows: *p* > 0.05: NS; ∗*p* ≤ 0.05; ∗∗*p* ≤ 0.01; ∗∗∗*p* ≤ 0.001; ∗∗∗∗*p* ≤ 0.0001. The detailed statistical analyses of the data are available in Table S3.

Previous studies of hemizygous *Hq* mice (*Hq*/Y) demonstrated that these mice display growth delay.^35^ We also observed that body weights of *Hq* mice increased at a slower rate than healthy mice of the same age.^39^ We evaluated 8-month-old *Hq* and control mice subjected to gene therapy in their cerebella 6 months earlier (Figure 1A). No change in the growth rate, as estimated by the body weight, was observed in control mice treated with AAV2/9-*GFP*, AAV2/9-*Aifm1*, or AAV2/9-*Ngb*: body weights attained 89.83% and 93.32% in *Aifm1*- or *Ngb-*treated mice relative to *GFP*-treated mice (*p* = 0.11 or 0.20, respectively).

Moreover, gene therapy did not exacerbate growth retardation in *Hq* mice. Body weights of *Hq* mice treated with AAV2/9-*GFP* reached 0.56-fold the ones assessed in control mice treated with the same vector (*p* < 0.0001). When the treatment was performed with AAV2/9-*Aifm1* or AAV2/9-*Ngb*, body weights of *Hq* mice reached 0.655- or 0.591-fold of the values assessed in control mice treated with the same vectors (*p* < 0.0001 for both vectors). So, comparisons of body weights between *Hq* mice treated with AAV2/9-*GFP* and *Hq* mice treated with either AAV2/9-*Aifm1* or AAV2/9-*Ngb* were not statistically different (*p* = 0.999 or 0.984). Thus, the growth delay in *Hq* mice subjected to gene therapy had the same extent whatever vector was administered.

Next, the weights of dry cerebella were compared between control and *Hq* mice treated with either one of the three vectors by calculating the ratios of cerebellar weights to body weights. We observed that *Hq* mice treated with AAV2/9-*Aifm1* or AAV2/9-*Ngb* exhibited heavier cerebella relative to *Hq* mice treated with AAV2/9-*GFP*.

The increase in tissue weight is illustrated in Figure 1B, when the ratios of cerebellar weight against body weight were measured. The ratio for *GFP*-treated *Hq* reached only 0.60 relative to their control counterparts (*p* = 0.065). Conversely, *Aifm1*-treated *Hq* and *Ngb*-treated *Hq* mice exhibited ratios of 1.19 and 1.30 relative to control mice treated with the same vectors (*p* = 0.673 and *p* = 0.145). Gene therapy with either *Aifm1* or *Ngb* increased cerebellar to body weight ratios by 71.4% and 200.7% when compared with *GFP*-treated *Hq* mice (*p* = 0.014 or *p* = 00009 for AAV2/9-*Aifm1* or AAV2/9-*Ngb*, respectively). Thus, *Aifm1* or *Ngb* overexpression partially counteracts the deleterious consequences for cerebellar degeneration of AIF depletion in *Hq* cerebella (Figure 1B).

### Purkinje cells were efficiently transduced by AAV2/9 vectors

**Figure 2:**
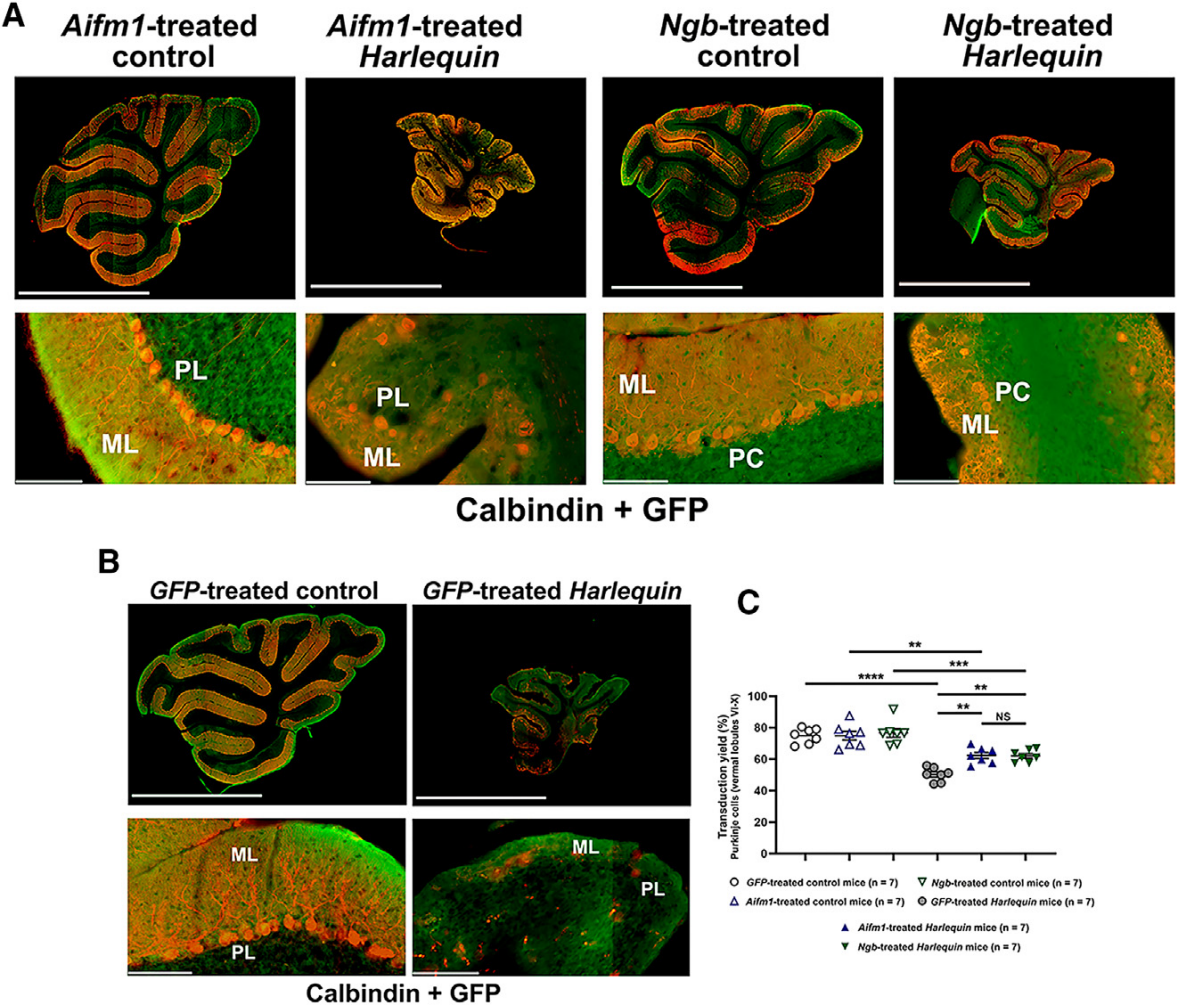
Gene therapy to target cerebellar neurons in *Harlequin* mice (A and B) Sagittal floating sections (40 μm) were prepared using a freezing microtome and stained with antibodies against calbindin (red) associated with antibody against GFP (green). The reconstruction of cerebellar sections obtained with the NanoZoomer Digital Pathology 2.0 HT scanner are illustrated for control mice treated with AAV2/9-*GFP*, AAV2/9-*Aifm1*, or AAV2/9-*Ngb* and *Hq* mice treated with AAV2/9-*GFP*, AAV2/9-*Aifm1*, or AAV2/9-*Ngb*; scale bars correspond to 2.5 mm. For each mouse, a higher magnification within lobule 9 was also shown (scale bar, 100 μm). The entire cerebellar area of the sections illustrated was calculated with ImageJ (in mm^2^) taking as reference the CALB labeling for *GFP-*treated *Harlequin*, 2.146; *GFP-*treated control, 8.622; *Aifm1*-treated *Harlequin*, 4.033; *Aifm1*-treated control, 8.522; *Ngb*-treated *Harlequin*, 3.934; *Ngb*-treated control, 8.46. ML, molecular layer; PL, Purkinje cell layer; PC, Purkinje cells. The concentrations of the primary and secondary antibodies used for immunohistochemistry are shown in Table S1. (C) The total number of Purkinje cells (Calbindin-positive cells), which were also stained with the GFP antibody, were counted in the posterior lobules (VI to X) for up to eight independent slices per mouse, this number is considered as the transduction yield of Purkinje cells in this region. Each bar corresponds to the means ± SEM obtained for each mouse group studied; their numbers are indicated in brackets (legend for each group). The overall data have been processed with GraphPad Prism 10.2. software and compared with the two-way ANOVA test (normal distribution of the data). The detailed statistical analyses of the data are available in Table S3.

To assess the transduction yield of Purkinje cells, sections of median region of the cerebellum (vermis) were submitted to double immunostaining against GFP and calbindin (CALB), a reliable marker of Purkinje cells.^40^ Figure 2A illustrates cerebellar reconstructions from *Hq* and control mice treated with AAV2/9-*Aifm1* or AAV2/9-*Ngb* and euthanized 6 months later. Two magnifications are shown: lower magnification to visualize the entire tissue, and a higher magnification of lobule IX to have a better view of the Purkinje cell layer (PL) within this lobule. Signal intensities of GFP labeling indicate that many neurons were transduced within both the granular cell layer (GL) and PL in treated animals. Moreover, cerebella from *GFP*-treated *Hq* mice showed smaller cerebella with shrunken cell layers; while some extent of preservation in cerebellar morphology was observed in *Hq* mice treated with either AAV2/9-*Aifm1* or AAV2/9-*Ngb* vector (Figures 2A and 2B).

The number of CALB-positive cells that were also labeled with the antibody against GFP in lobules VI to X (posterior part of the cerebellum) were counted in seven *Hq* or seven control mice treated with either one of the vectors. Transduction yields in control mice were not significantly different regarding the administered vectors (*p* > 0.9999): 74.85%, 74.91%, and 76.64% of Purkinje cells were transduced when control mice were treated with AAV2/9-*Aifm1*, AAV2/9-*Ngb*, and AAV2/9-*GFP* vectors (Figure 2C). On the contrary, there was a significant difference between treated *Hq* mice depending on the administered vector: the percentage of transduced cells in *GFP*-treated *Hq* mice was 50.18%; while it reached 62.4% and 62.1% in *Aifm1*-and *Ngb*-treated *Hq* mice. Thus, an increase of about 24% of transduced cells was observed in cerebella that overexpressed *Aifm1* or *Ngb*, the difference with *GFP*-treated *Hq* was significant (*p* values were respectively 0.0048 and 0.0063; Figure 2C). When the comparison was performed between control and *Hq* mice, a reduction of 33% in transduction yield was evidenced in AAV2/9-*GFP*-treated *Hq* mice relative to *GFP*-treated control mice (*p* < 0.0001). On the contrary, the reduction in *Hq* mice treated with AAV2/9-*Aifm1* or AAV2/9-*Ngb* relative to their control counterparts was minor, e.g., 17% and 19%; the difference becoming less significant between *Hq* and control mice; *p* = 0.0035 and 0.0006 (Figure 2C).

The differences observed between control and *Hq* mice could be explained by the delay of about 4 weeks required to reach the maximum level of proteins synthesized from recombinant vectors.^41^

**Figure 3:**
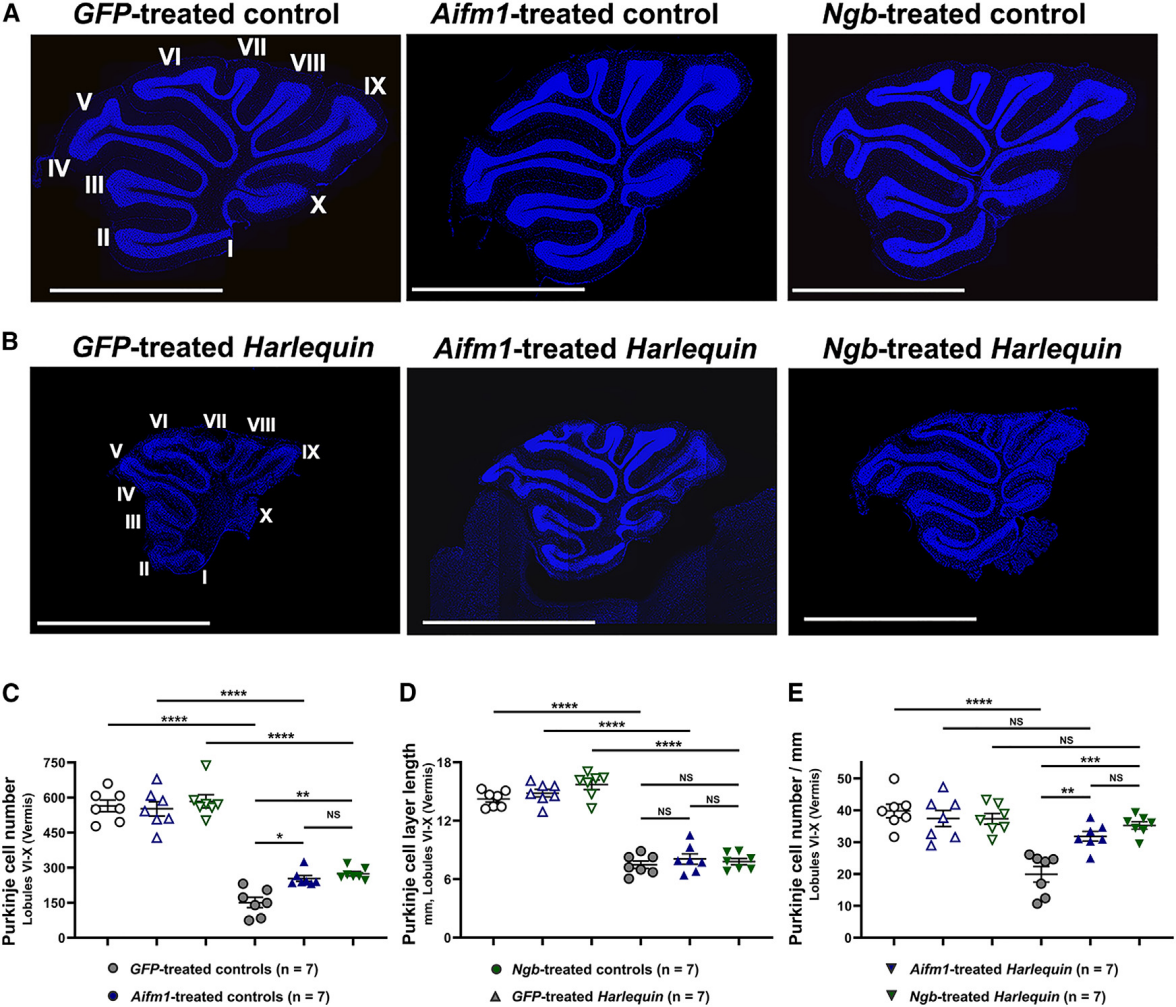
Neuronal cell loss in cerebella from *Harlequin* mice (A) The reconstruction of cerebellar sections was obtained in scanned sections. DAPI labeling of nuclei (blue) is shown for one *GFP*-treated control mouse, one *GFP*-treated *Hq* mouse, one *Aifm1*-treated control mouse, one *Aifm1*-treated control mouse, one *Ngb*-treated control mouse, and one *Ngb*-treated *Hq* mouse. Each lobule is numbered from I to X in the images corresponding to the cerebellum from a control mouse and a *Harlequin* mouse (left side of the figure). The scale bars correspond to 2.5 mm. (B) Calbindin-positive cells were counted in reconstructed sections scanned with the NanoZoomer scanner using the NDP.view2 software. (C) The total number of Purkinje cells, labeled with the antibody against calbindin, was determined in the posterior lobules (VI to X) for up to six independent slices per mouse (left panel). (D) The total length of the Purkinje cell layer was calculated in the posterior part of the tissue between lobules VI to X a for up to six independent slices per mouse and is expressed in mm. (E) The number of Purkinje cells was normalized against the total length (mm) of the Purkinje cell layer between lobules VI to X (right panel). Histograms illustrate the means of values ± SEM for control and *Hq* mice, 6 months after vector administration. The numbers of mice evaluated for each vector are indicated in brackets.To compare the different groups, GraphPad Prism 10.2 software was used. The *p* values were determined by applying the Kruskal-Wallis test for Purkinje cell numbers since the groups deviated from a Gaussian distribution. For Purkinje cell layer length and the Purkinje cell number per mm, the groups were compared with the two-way ANOVA test (data followed a Gaussian distribution). *p* values in each histogram are as follow: *p* > 0.05: NS (not significant); ∗*p* ≤ 0.05; ∗∗*p* ≤ 0.01; ∗∗∗*p* ≤ 0.001; ∗∗∗∗*p* ≤ 0.0001. The detailed statistical analyses of the data are available in Table S3.

Additional reconstructions of cerebellar sections from 8-month-old mice subjected to gene therapy are shown in Figure 3 to get a better overview of nuclei in the different cell layers within the vermis. Cerebella from control mice were very similar independent of the vector used (Figure 3A). In *Ngb*- or *Aifm1*-treated *Hq* mice, a higher fluorescent intensity was observed relative to *GFP*-treated *Hq* mice. This supports the assumption that the extent of neuronal cell survival increased in cerebella from *Hq* mice, which overexpressed *Aifm1* or *Ngb*. In addition, the structure and size of individual lobules appeared to be better preserved in *Aifm1*- or *Ngb*-treated *Hq* mice than in *GFP*-treated *Hq* mice (Figure 3B).

Next, the number of Purkinje cells in the vermal lobules VI to X was determined in the six groups of mice subjected to gene therapy (Figure 3C). First, Purkinje cell numbers were compared in control mice treated with AAV2/9-*GFP*, AAV2/9-*Aifm1*, or AAV2/9-*Ngb*; no significant changes in their numbers were evidenced (*p* > 0.9999). *Hq* mice treated with AAV2/9-*GFP* exhibited a 73.2% diminution when compared with control mice treated with AAV2/9-*GFP* (*p* < 0.0001). Though, cerebella from treated *Hq* mice with AAV2/9-*Aifm1* or AAV2/9-*Ngb* displayed a smaller decrease of Purkinje cell number relative to the values found in their control counterparts: 53.9% or 53.1% for AAV2/9-*Aifm1* or AAV2/9-*Ngb* (*p* < 0.0001 for both treatments). Moreover, cerebella from *Hq* mice overexpressing *Aifm1* or *Ngb* exhibited respectively 68.2% or 81.3% more Purkinje cells than those from *GFP-*treated *Hq* mice (*p* = 0.020 or 0.0046, respectively). Consequently, *Aifm1* or *Ngb* overexpression in the cerebellum of *Hq* mice allowed a partial preservation of Purkinje cells. The normalization of Purkinje cell number was achieved by estimating the length of the Purkinje cell layer between lobules VI and X. It is noticeable that the length of the Purkinje cell layer was reduced by 47% in *GFP*-treated *Hq* mice compared with *GFP*-treated control mice, *p* < 0.0001 (Figure 3D). On the other hand, the length of the Purkinje cell layer in *Aifm1*-treated *Hq* or *Ngb*-treated-*Hq* mice was low and comparable to the one measured in *GFP*-treated *Hq* mice (*p* = 0.91 and 0.99, respectively). Next, the Purkinje cell number per millimeter of each cerebellum was compared between treated *Hq* and control mice (Figure 3E). When *GFP*-treated *Hq* and *GFP*-treated control mice were compared, a diminution of 50.1% was evidenced (*p* < 0.0001). Overexpression of *Aifm1* or *Ngb* largely attenuated this diminution, indeed the mean values of Purkinje cell number per millimeter of Purkinje cell layer length attained 85.1% and 94.5% of the values measured in their control counterparts treated with the same vectors. Consequently, for this parameter there was no more significant difference between *Hq* mice and their control counterparts (*p* = 0.45 or 0.98, respectively, for *Aifm1* or *Ngb*). Purkinje cell number per millimeter was superior by 59.7% or 76.9% in cerebella overexpressing *Aifm1* or *Ngb* when compared with those found in *GFP*-treated *Hq* cerebella (*p* = 0.0053 or 0.0002 for *Aifm1* or *Ngb*). Moreover, there was no statistical difference between cerebella from *Hq* mice that overexpressed *Aifm1* or *Ngb* (*p* = 0.86); thus, the ability of the corresponding proteins to protect Purkinje cells was similar.

### Abundance of NGB and AIF in cerebella subjected to gene therapy

**Figure 4:**
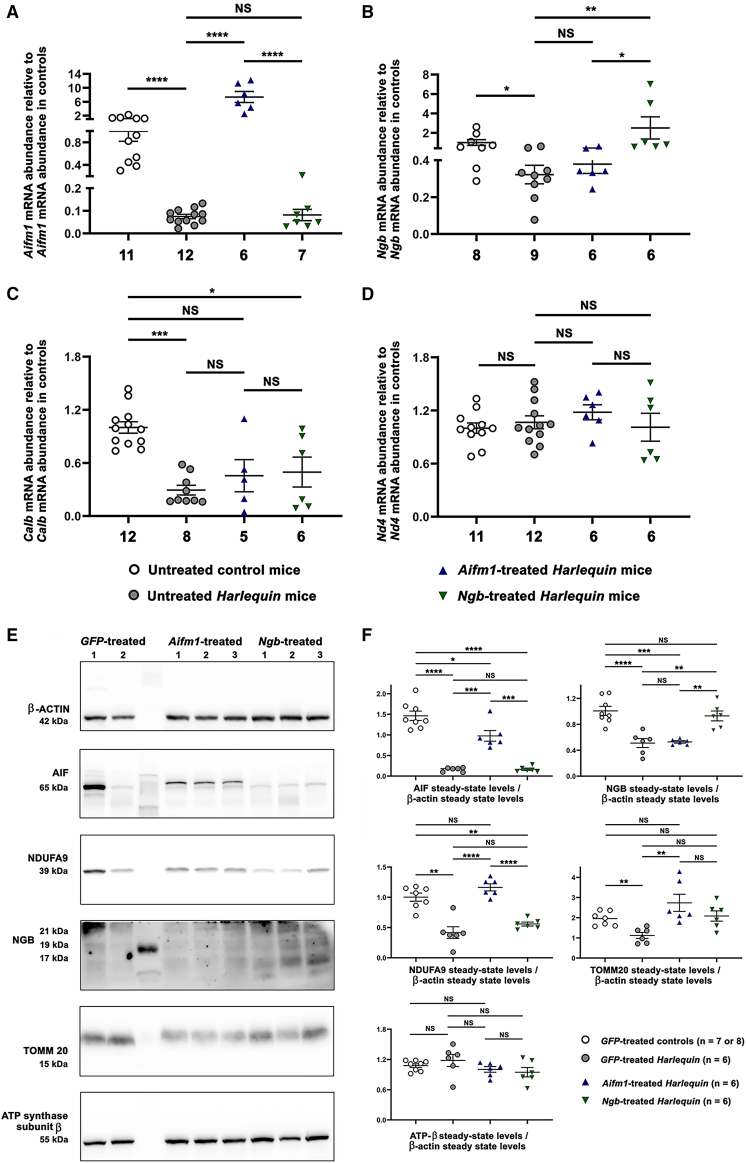
Relative abundance of neuroglobin and apoptosis-inducing factor in treated cerebella To estimate the steady-state levels of *Ngb* or *Aifm1* mRNA, qPCR assays were performed using total RNAs isolated from cerebella of untreated control mice and *Hq* mice aged ∼8 months as well as *Hq* mice treated with either AAV2/9-*Aifm1* and AAV2/9-*Ngb* at the age of 2 months and euthanized 6 months later. The number of RNA samples assessed is indicated below each bar of the histograms. The steady-state levels of the following mRNAs were measured: *Aifm1*, *Ngb*, calbindin (*Calb*), and *Nd4*; this latter is transcribed from the mitochondrial DNA and encodes the subunit 4 of respiratory chain complex I (NADH:ubiquinone oxidoreductase).Steady-state levels of *Aifm1* mRNA (A), *Ngb* mRNA (B), *Calb* mRNA (C), and *Nd4* mRNA (D) are shown as means ± SEMs after normalization against the mean signals for *Aifm1*, *Ngb*, *Calb*, or *Nd4* mRNA measured in cerebellar preparations from untreated control mice. Statistical analyses were performed with the GraphPad Prism 10.2 software. The *p* values shown were calculated with respect to data collected from untreated control mice. Specific primers used for *Aifm1*, *Ngb*, *Calb*, and *Nd4* mRNAs are shown in Table S2. (E) Representative images obtained when whole-protein extracts (30 μg) from *Hq* and control cerebella were subjected to western blot analyses; mice were euthanized at the age of 8 months. Proteins isolated from eight *GFP*-treated controls, six *GFP*-treated *Hq* mice, six *Hq* mice treated with the AAV2/9-*Aifm1*, and six *Hq* mice treated with the AAV2/9-*Ngb* vector were evaluated. The membranes were successively incubated with antibodies against neuroglobin (NGB), apoptosis-inducing factor (AIF), TOMM 20, ATP synthase subunit β, NDUFA9, and β-actin (as the loading control). Protein extracts from one *GFP*-treated control and one GFP-treated *Hq* mouse are shown in the left part of the image. Next, results for three cerebella from *Hq* mice injected with AAV2/9-*Aifm1* and three cerebella of *Hq* mice injected with AAV2/9-*Ngb* are shown; all these samples run in the same SDS gel. Automatic molecular weight calculations for each band using GeneTools from Syngene are mentioned below each image: 65 kDa for AIF, 39 kDa for NDUFA9, 15 kDa for TOMM 20, 55 kDa for subunit β of ATP synthase and 42 kDa for β-actin, measures close from their theoretical molecular masses. The antibody against NGB yielded two main signals, with apparent molecular masses of 21 kDa, 19 kDa, and 17 kDa. The concentrations of the primary and secondary antibodies used are shown in Table S1. (F) Bar charts show the relative amounts of each of these proteins after the normalization of their signals against β-actin signal in cerebella from *GFP*-treated control mice, *GFP*-treated *Hq* mice, and cerebella isolated from *Aifm1*-or *Ngb*-treated *Hq* mice. The number of independent cerebella evaluated is shown for each group in the legend (bottom-right); each sample was run at least three times. The histograms were obtained using the means ± SEM for each sample. The *p* values shown were calculated with the GraphPad Prism 10.2 and detailed statistical analyses are available in Table S3.

To determine the changes in steady-state levels of AIF and NGB as a result of gene therapy, we first measured the level of *Aifm1* and *Ngb* mRNAs (qPCR) with RNA preparations from *Hq* cerebella treated with AAV2/9-*Ngb* or AAV2/9-*Aifm1* vector and from cerebella from 8-month-old untreated control or *Hq* mice (Figure 4). The amount of *Aifm1* mRNA in cerebella from untreated *Hq* mice was only 0.075 times the amount measured in untreated controls. AAV2/9-*Aifm1* administration in the cerebella from *Hq* mice led to a 7.4-fold increase in the amount of *Aifm1* mRNA relative to those measured in cerebella from control mice (*p* < 0.0001) and a 98.4-fold increase relative to the amount measured in cerebella from untreated *Hq* mice, *p* < 0.0001 (Figure 4A). However, *Aifm1* mRNA level remained unchanged in *Ngb*-treated cerebella relative to that in untreated *Hq* tissues (*p* = 0.499).

Steady-state levels of *Ngb* mRNA attained only 32.3% of the value measured in cerebella from untreated control mice (*p* = 0.044). In cerebella from *Ngb*-treated *Hq* mice, there was a 7.8-fold increase in *Ngb* mRNA amount relative to those found in cerebella from untreated *Hq* mice (*p* = 0.005) and a 2.5-fold increase when compared with the value measured in cerebella from untreated control mice, values were no more statistically different, *p* = 0.223 (Figure 4B). Besides, the endogenous expression of *Ngb* remained unchanged in *Aifm1*-treated cerebella when compared with the one in untreated *Hq* cerebella, *p* > 0.999 (Figure 4B). We also observed a decrease of 70% in the amount of *Calb* mRNA in cerebella from untreated *Hq* mice relative to control mice (*p* = 0.0005) likely due to the disappearance of Purkinje cells. An increase of 55.6% and 69.6% in its steady-state levels was observed in *Aifm1*-treated and *Ngb*-cerebella from *Hq* mice; thus, the difference between *Calb* mRNA levels in treated *Hq* and control mice became less significant (*p* = 0.066 or 0.039, respectively). *Nd4* mRNA amount, which is transcribed from the mitochondrial genome, was unchanged in all mouse groups examined (Figure 4D).

We then assessed *Aifm1* and *Ngb* expression at the protein level in cerebellar homogenates by western blots and after normalization against the signal obtained with β-actin antibody (Figures 4E and 4F). In the cerebellum of *GFP*-treated *Hq* mice, AIF or NGB level was only 10.9% (*p* < 0.0001) or 51.1% (*p* < 0.0001) relative to *GFP*-treated control mice. As a result of gene therapy, cerebella from *Aifm1*-treated *Hq* or *Ngb*-treated *Hq* accumulated significantly more of AIF (66.7%) or NGB (92.4%) relative to *GFP*-treated control mice (*p* = 0.011 and 0.47, respectively). Moreover, the steady-state levels of AIF and NGB were 6.15-fold and 1.82-fold more significant in treated *Hq* cerebella than in *GFP*-treated *Hq* mice (*p* = 0.0002 and 0.0013, respectively; Figures 4E and 4F). These values are reminiscent of that it was observed for mRNA abundance (Figures 4A and 4B) and confirmed the effectiveness of vector transduction of cerebellar neurons from *Hq* mice.

The absence of AIF in *Hq* cerebella resulted in a 56.6% reduction of NDUFA9 (complex I component) abundance relative to cerebella from control mice (*p* = 0.0001); in line with previous reports in other mouse tissues or AIF-depleted cells.^42^ In contrast, *Aifm1* overexpression in *Hq* cerebella led to a 2.79-fold increase of NDUFA9 abundance relative to *GFP*-treated *Hq* mice (*p* < 0.0001), while the amount of NDUFA9 in *Hq* samples overexpressing *Ngb* was not different from those observed in untreated *Hq* mice, *p* = 0.397 (Figures 4E and 4F).

Interestingly, the amount of TOMM20 (a protein of the outer mitochondrial membrane) decreased by 42.6% in *GFP*-treated *Hq* cerebella relative to *GFP*-treated control cerebella (*p* = 0.0014). The amount of TOMM 20 in *Aifm1*-treated *Hq* cerebella attained 140% of the amount in control samples; hence, these two groups became similar (*p* = 0.261). For *Ngb*-treated *Hq* samples, an increase of TOMM 20 was also observed, but at a lesser extent than in mice that received AAV2/9-*Aifm1*; indeed, cerebella accumulated as much of TOMM 20 than *GFP*-treated controls and the two groups were not different (*p* = 0.98; Figures 4E and 4F). Finally, steady-state levels of ATP synthase subunit β, a subunit of respiratory complex V, were similar in all the groups evaluated (Figures 4E and 4F).

**Figure 5:**
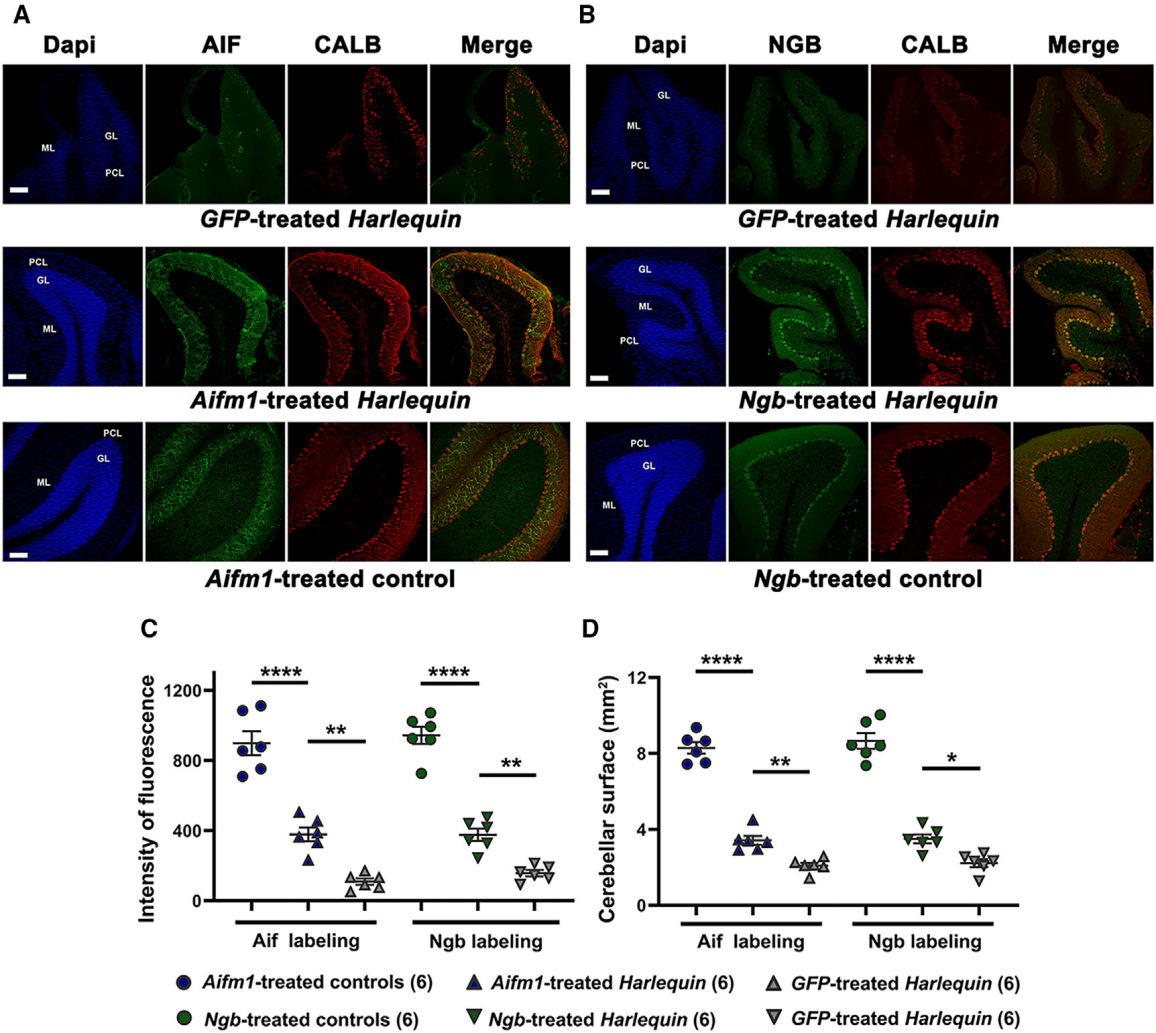
Morphological analysis of cerebellar sections from *Harlequin* and control mice (A and B) Sagittal sections (40 μm) were prepared from control or *Hq* mice treated with either AAV2/9-*Aifm1*, AAV2/9-*Ngb*, or AAV2/9-*GFP* euthanized 6 months post-treatment and stained with the antibody against AIF or NGB combined with the antibody against calbindin (Calb). Illustrated are fluorescent signals for Ngb or AIF protein (green), calbindin (red), and the overlaying of the two labeling (Merge) for treated mice and two untreated *Hq* mice aged ∼8 months. DAPI was used to visualize all the nuclei (blue). Images were obtained from Leica TCS SP8 confocal microscope and the LAS X software; scale bars correspond to 100 μm (20× objective with a 0.75× zoom). The concentrations of the primary and secondary antibodies used for immunohistochemistry are shown in Table S1. (C) and (D) The intensity of AIF or NGB in order to be in agreement of the order (C) and (D) staining in cerebellar sections and their overall area in mm^2^ for NGB- or AIF-labeled slides were estimated with the ImageJ software using reconstructed cerebellar sections generated by the NDP 2.0 HT scanner and exported as tiff images. Values were plotted as means ± SEMs, the number of mice analyzed is indicated below each bar of the histograms. Reports of statistical significance, *p* values, from GraphPad Prism in each histogram are as follows: *p* > 0.05: NS (not significant); ∗*p* ≤ 0.05; ∗∗*p* ≤ 0.01; ∗∗∗*p* ≤ 0.001; ∗∗∗∗*p* ≤ 0.0001. The detailed statistical analyses of the data are illustrated in Table S3.

To determine whether the subcellular localization of NGB or AIF may be altered as a result of *Ngb* or *Aifm1* overexpression, immunohistochemistry was performed in cerebellar sections with antibodies against two mitochondrial proteins ND6 (a subunit of respiratory chain complex I) or ATP β. First, cerebellar sections were labeled with antibodies against ND6 and ATP β to visualize in Purkinje cells the distribution of these two mitochondrial proteins that localize to the inner mitochondrial membrane (Figure S2A). In cerebellar sections from the untreated control mouse shown, immunoreactivity of antibodies revealed a punctuated distribution of fluorescent dots excluded from the nuclei that certainly correspond to mitochondria, a substantial extent of colocalization between the two proteins is noticed in the merge image as yellow-orange pixels (left panel). Next, Figures S1B and S1C illustrate confocal images of cerebellar sections from two untreated control mice and two untreated *Hq* mice, as well as one *Hq* mouse treated with AAV2/9-*Aifm1* and another one treated with AAV2/9-*Ngb* when the combinations of the following antibodies were carried out: ND6 + AIF or NGB + ATP β. Major observations can be made: (1) untreated *Hq* cerebella display a significant diminution of AIF and NGB amounts; (2) the fluorescent signal of the antibody against ATP β in the untreated *Hq* cerebellar section (Figure S2C) indicated that mitochondria in the soma of Purkinje cells exhibit morphological alterations; (3) the amount of ND6 is very low in untreated *Hq* mice, moreover, *Aifm1* overexpression results in a noticeable increase of ND6 abundance (Figure S1B) as we observed for NDUFA9 in western blot assays (Figures 4E and 4F); (4) the abundance of AIF or NGB was significantly increased in *Hq* mice treated respectively with AAV2/9-*Aifm1* or AAV2/9-*Ngb* vector relative to untreated *Hq* mice as evidenced by western blotting (Figures 4E and 4F); (5) the extent of colocalization and the characteristics of the labeling for AIF and ND6 or NGB and ATP synthase subunit β, evidenced as punctuate fluorescent dots excluded from the nuclei (yellow-orange pixels, merge images), supports the idea that overexpression of these genes in *Hq* cerebella did not change the subcellular localization of the corresponding proteins; and (6) according to immunohistochemistry using antibodies against ATP β and NGB, the morphology of mitochondria in *Ngb*-treated *Hq* mice appeared more organized and analogous to the one observed in control mice, as if gene therapy with AAV2/9-*Ngb* vector reduced changes of the organelle structure.

### Morphological evaluations of cerebella from *Hq* and control mice subjected to gene therapy

To better evaluate cerebellar structure after gene therapy, tissue sections from control and *Hq* mice treated with AAV2/9-*Aifm1*, AAV2/9-*Ngb*, or AAV2/9-*GFP* were subjected to immunohistochemistry using antibodies against AIF or NGB combined with the antibody against calbindin (CALB). The fluorescence intensity of the AIF or NGB labeling was enhanced in *Aifm1*-treated or *Ngb*-treated mice, respectively (Figures 5A and 5B), which corroborates qPCR and western blot analyses (Figure 4). ImageJ software was used to determine the overall fluorescent intensity of each antibody signal in cerebellar reconstructions from scanned slices (Figure 5C, left panel). The intensity of AIF signal in *GFP-*treated *Hq* mice relative to *Aifm1*-treated control mice was of 12.2% (*p* < 0.0001). In contrast, AIF signal in cerebella from *Aifm1*-treated *Hq* mice attained 42.2% of the signal obtained in *Aifm1*-treated controls, a 3.3-fold increase relative to *GFP*-treated *Hq* mice (*p* = 0.0075). For NGB, it was observed an overall intensity in *GFP-*treated *Hq* mice relative to *Ngb*-treated control mice of 16.7%; conversely, *Ngb*-treated *Hq* mice displayed an intensity equivalent of 39.9% of the one measured in *Ngb*-treated control mice, an increase of 2.4-fold relative to *GFP*-treated *Hq* mice (*p* = 0.0012).

Next, cerebellar areas were calculated with ImageJ by applying the Huang algorithm to automatically delimit and calculate the total cerebellar area (mm^2^) for either AIF or NGB immunolabeling. In *Ngb*- or *Aifm1*-treated *Hq* mice, cerebellar surface was higher by ∼25% relative to *GFP*-treated *Hq* mice (Figure 3C, right panel). The difference was statistically significant when *Ngb*- or *Aifm1*-treated *Hq* mice were compared with *GFP*-treated *Hq* mice since the increase of the area was of 64.1% and 57.3% for *NGB*- and *Aifm1*-treated *Hq* mice (*p* = 0.027 or 0.0037, respectively). Thus, the diminution regarding tissue area between treated controls and treated *Hq* was 41.3% and 40.4% instead of 64.1% for *GFP*-treated *Hq* mice. These data indicate that gene therapy with AAV2/9-*Aifm1* or AAV2/9-*Ngb* yield a partial protection of neuronal cell population.

Subsequently, we proceeded to another immunohistochemical assay with cerebellar sections from seven *Hq* mice treated with AAV2/9-*Aifm1*, seven *Hq* mice treated with AAV2/9-*Ngb,* seven *Hq* mice treated with AAV2/9-*GFP*, nine control mice, and 10 *Hq* mice aged 8 months that remained untreated (Figure S3). β3-Tubulin is one of the microtubule cytoskeleton components involved in multiple essential functions during neurodegeneration.^43^ Its use allows the visualization of somas, dendrites, and axons of Purkinje cells. Imaging with the confocal microscope using a high magnification indicated that in untreated *Hq* mice not only Purkinje cells (calbindin-positive cells, in red on the images) were reduced in number but also that their dendritic arborizations were scarce and disorganized. On the other hand, treated *Hq* mice displayed more Purkinje cells with a better preserved dendritic arborization through the molecular cell layer when compared with untreated *Hq* mice. Next, we determined the molecular layer thickness, which contains the extended and elaborate dendrites of Purkinje cells. We chose to measure, in the vermis, the thickness of lobule IX; this lobule is engaged along with lobule VI in tasks of visuospatial memory,^44^ nonverbal working memory,^45^ and in higher cognitive circuitry with lobules VII and part of lobule VI.^46^^,^^47^

We assume that this metric is an accurate indicator of Purkinje cell arborization complexity that correlates with their functionality. We observed a 58.7% reduction of lobule IX molecular layer thickness in untreated *Hq* mice relative to age-matched controls; while the molecular layer thickness was significantly increased by 64.5% or 64.4% after gene therapy with AAV2/9-*Aifm1* or AAV2/9-*Ngb* relative to *GFP*-treated *Hq* mice (*p* < 0.0001 for each vector). Hence, the administration of AAV2/9-*Aifm1* or AAV2/9-*Ngb* in the cerebella of *Hq* mice resulted in a preserved connectivity of the remaining Purkinje neurons (Figure S3B). These data corroborate the benefit of *Aifm1* or *Ngb* overexpression for the maintenance of cerebellar morphology in *Hq* mice.

### Gene therapy mitigates mitochondrial alterations in the cerebellum of *Hq* mice

**Figure 6:**
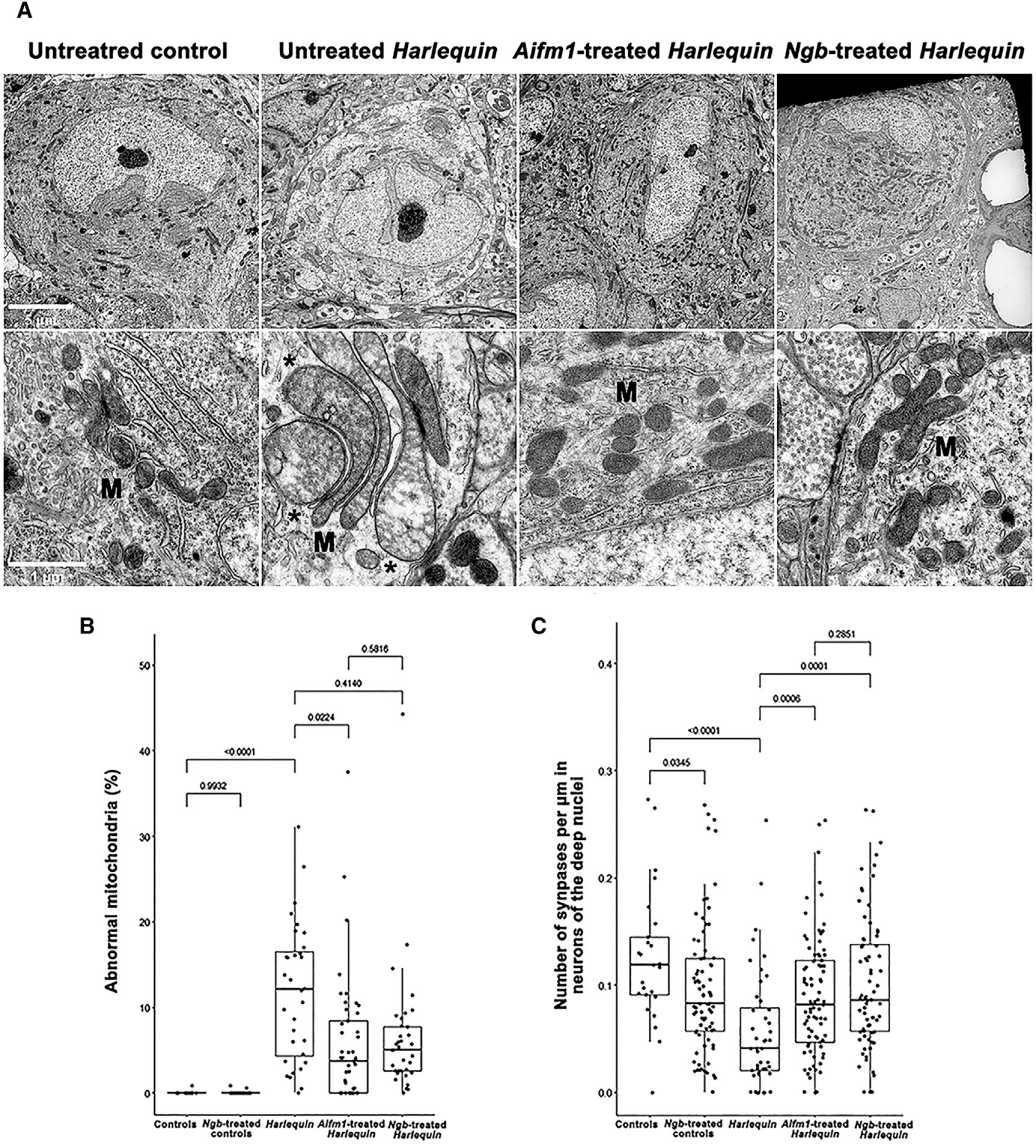
Ultrastructural changes in Purkinje cells from *Harlequin* mice subjected to gene therapy (A) Purkinje cells in untreated mice aged 8 months (control and *Hq*), one *Aifm1*-treated *Hq* mouse, and one *Ngb*-treated *Hq* mouse (from left to right in the figure). The top part shows Purkinje cell soma at low magnification (scale bar, 5 μm). The bottom part shows a zoom at high magnification (scale bar, 1 μm) to better assess the ultrastructure within the Purkinje cell soma of mitochondria (M); ∗ illustrates swollen mitochondria in untreated *Hq* mice (bottom part of the image). The following are the groups of mice examined: untreated control mice, 2; untreated *Harlequin* mice, 4; *Ngb*-treated control mice, 5; *Aifm1*-treated *Harlequin* mice, 5; *Ngb*-treated *Harlequin* mice, 5. (B) The histogram shows the percentage of highly swollen mitochondria (Category 4) in each mouse group; the number of Purkinje cell somas examined is indicated under each column. The comparison of each mouse group (by pairs) was performed using the unpaired Mann-Whitney test. (C) The histogram illustrates the number of synapses made between Purkinje cell axons and neurons located in the median deep nuclei per μm of length after the calculation of the neuron circumference (μm). The number of synapses counted is indicated under each column. Statistical evaluation was performed with GraphPad Prism; the detailed statistical analyses for each set of the data are available in Table S3.

In *Hq* mice, Purkinje cells are characterized by an extensive degeneration of their mitochondria.^39^ Thus, we assessed the impact of gene therapy for mitochondrial ultrastructural alterations using transmission electron microscopy (TEM). The study was conducted in the lobule IX of the cerebellum. Figure 6A shows representative micrographs of Purkinje cell somas for each group studied. In Purkinje cells from untreated control mice, mitochondria displayed clearly visible cristae in a moderately dense matrix without any sign of swelling. In contrast, almost all Purkinje cells in untreated *Hq* mice displayed alterations such as cristae disappearance, swelling, thinning, and stacking. Interestingly, in *Aifm1*-treated and *Ngb*-treated *Hq* mice, PCs were often found with fewer abnormal mitochondria. Using high-resolution micrographs of individual neurons, we quantitatively assessed the extent of the mitochondrial swelling. A total of 44 Purkinje cells from two to five mice per group were included in this analysis. In each cell, all mitochondria were counted and the proportion of highly swollen mitochondria was determined. As shown in Figure 6B, the percentage of highly swollen mitochondria in Purkinje cells was very small in untreated and *Ngb*-treated control mice (0.055% and 0.033%, respectively) but increased by 11.76%, a 211.77-fold in untreated *Hq* mice relative to untreated controls (*p* < 0.0001). *Aifm1* or *Ngb* overexpression significantly reduced this proportion to respectively 101.73- and 117.39-fold relative to untreated control mice. The extent of abnormal mitochondria in cerebella which overexpressed *Aifm1* or *Ngb* was reduced by 52.0% or 44.6% relative to the amount in untreated *Hq* (*p* < 0.0001 for *Aifm1* and *p* = 0.0004 for *Ngb*). The percentage of highly swollen mitochondria was not different between cerebella that overexpressed *Aifm1* or *Ngb* (*p* = 0.217). Purkinje cells extend a single axon that travels through the GL before making both axo-somatic and axo-dendritic synapses onto the DCN. Using high-resolution TEM micrographs, we measured the linear density of axo-somatic synapses upon neuron soma in the median DCN (number of synapses from Purkinje cell axon terminals divided by the circumference of the soma expressed in μm). Figure 6C illustrates the results obtained: untreated *Hq* mice exhibited only 43.3% of synapses relative to the value measured in control mice (*p* < 0.0001). *Hq* mice treated with AAV2/9-*Aifm1* or AAV2/9-*Ngb* exhibited an increase of 71.3% or 77.9% in the linear density of axo-somatic synapses relative to untreated control mice. When untreated and treated *Hq* mice were compared, a 1.65-fold or 1.80-fold increase in *Aifm1*-treated *Hq* mice (*p* = 0.0006) or *Ngb*-treated *Hq* mice (*p* = 0.0001) were found. No significant difference was found between the two treatments (*p* = 0.285).

Considering these results, treatment of *Hq* mice with AAV2/9-*Aifm1* or AAV2/9-*Ngb* partially prevented the alteration of mitochondrial morphology in Purkinje cell somas and preserved the ability of their axons to make synapses onto their target neurons in the DCN.

### Gene therapy preserves the energy status of cerebella from *Hq* mice

**Figure 7:**
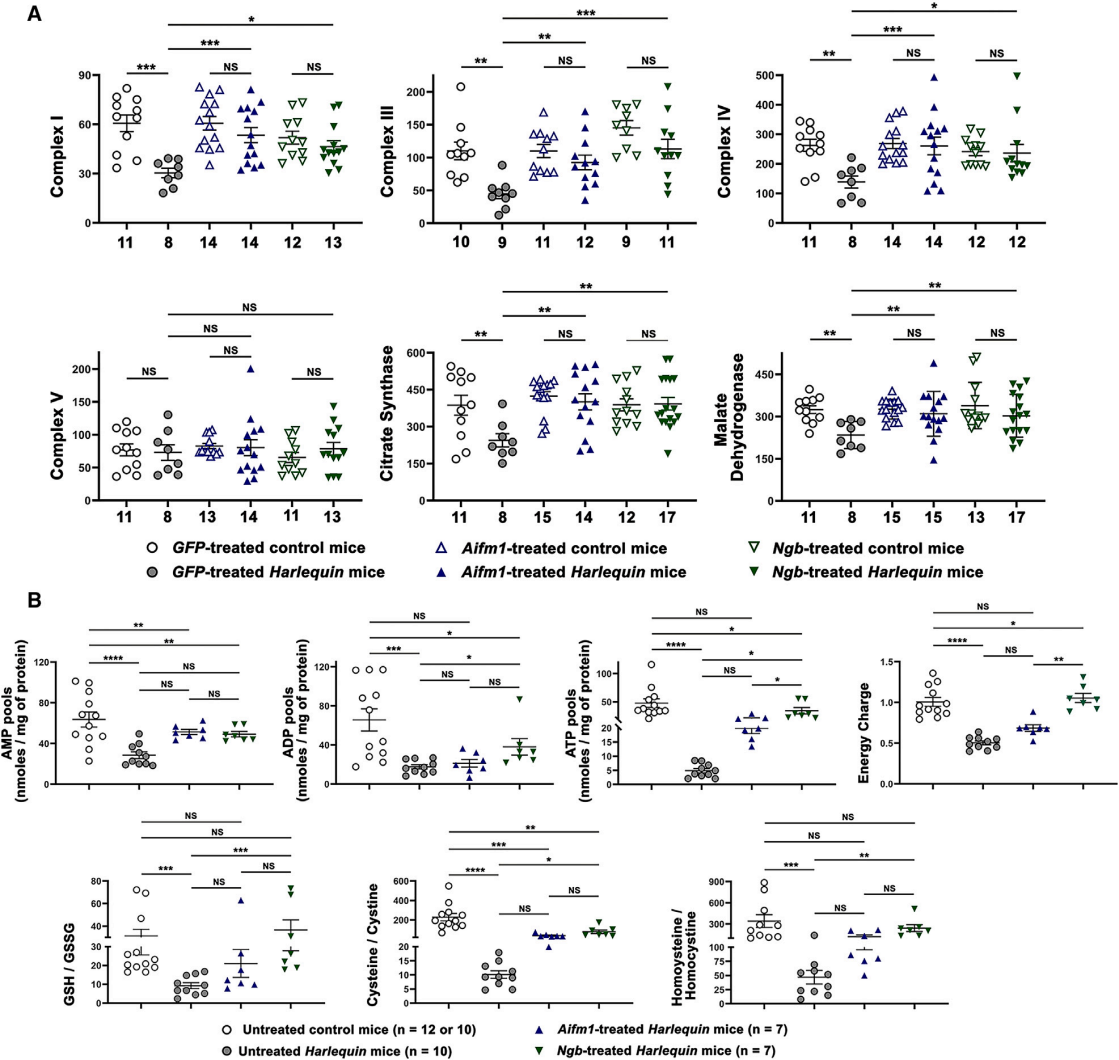
Bioenergetics status of cerebella from *Harlequin* mice subjected to gene therapy (A) The enzymatic activity of respiratory chain complex I, complex III, complex IV (upper panel), complex V, citrate synthase and malate dehydrogenase (bottom panel) were assessed in cerebella isolated from *Harlequin* and control mice euthanized 6 months after the administration of recombinant AAV2/9 vectors. Cerebellar homogenates from the following groups were assessed: (1) *GFP*-treated control mice; (2) *GFP*-treated *Harlequin* mice; (3) *Aifm1*-treated control mice; (4) *Aifm1*-treated *Harlequin* mice; (5) *Ngb*-treated control mice; and (6) *Ngb*-treated *Harlequin* mice. Histograms illustrate the enzymatic activities as means ± SEM of each assay per sample measured in triplicate as described in the material and methods section. The numbers of independent samples tested are indicated below each column. Complex I and complex V activities are expressed as nanomoles of oxidized NADH/min/mg protein; complex IV activity is expressed as nanomoles of oxidized cytochrome *c*/min/mg protein. The citrate synthase activity is expressed as nanomoles of reduced 5.5′Dithio-bis 2-Nitrobenzoic acid (DTNB)/min/mg protein. Malate dehydrogenase activity is expressed as nanomoles of oxidized NADH/min/mg protein. (B) Ultra-performance liquid chromatography coupled with tandem mass spectrometry (UPLC-MS/MS) was carried out to determine the content of nucleotides and the energy charge (EC) in cerebellar homogenates from untreated control and *Hq* mice aged 8 months, *Aifm1*-treated *Hq* mice, and *Ngb*-treated *Hq* mice. Thus, the upper panel shows adenosine 5′-monophosphate (AMP); adenosine 5′-diphosphate (ADP); adenosine 5′-triphosphate (ATP); and energy charge, EC, which was calculated using the following formula: (ATP + 0.5 ADP)/(ATP + ADP + AMP). UPLC-MS/MS allowed also to determine three biomarkers of oxidative stress in frozen cerebellar samples belonging to the same groups used in the upper panel. Accordingly, the amounts of GSH, GSSG, cysteine, cystine, homocysteine and homocysteine were measured and the corresponding ratios were calculated. Graphic representations of values obtained for each group correspond to the means ± SEM, the number of mice per group are indicated in the corresponding legend. The overall statistical analyses were performed using the GraphPad Prism 10.2 program. *p* values in each histogram are as follows: *p* > 0.05: NS (not significant); ∗*p* ≤ 0.05; ∗∗*p* ≤ 0.01; ∗∗∗*p* ≤ 0.001; ∗∗∗∗*p* ≤ 0.0001. The detailed statistical analyses for the data are available in Table S3.

As known, the main source of cellular energy is produced by OXPHOS within the mitochondria performed by the electron transport chain (complexes I-IV) and the ATP synthase (complex V) and also that neuronal survival, especially Purkinje cells, is dependent of the appropriate production of ATP and the functional integrity of mitochondria.^48^ Therefore, to determine whether the improvements of mitochondrial ultrastructure in Purkinje cells from *Hq* mice overexpressing *Aifm1* or *Ngb* enhanced their bioenergetics’ status of cerebellar neurons, enzymatic activities of respiratory chain complexes I (CI), III (CIII), IV (CIV), and V (CV) and of two proteins of the TCA cycle were measured by spectrophotometry. The comparison was made between cerebella from controls and *Hq* mice treated with AAV2/9-*GFP*, AAV2/9-*Aifm1*, or AAV2/9-*Ngb* (Figure 7A). Relative to *GFP*-treated control mice, the enzymatic activities of CI, CIII, and CIV were reduced respectively by 50.0% (*p* = 0.0008), 59.5% (*p* = 0.0011), and 47.1% (*p* = 0.0025) in *GFP*-treated *Hq* mice indicating a severe respiratory chain dysfunction. Gene therapy using either AAV2/9-*Aifm1* or AAV2/9-*Ngb* resulted in the restoration of enzymatic activities for the three complexes assessed: overexpression of *Aifm1* in *Hq* cerebella allowed activities to reach respectively 0.88, 0.84, or 0.967 of the values measured in controls treated with AAV2/9-*Aifm1* (*p* = 0.76, 0.96, or 0.99 respectively for CI, CIII, or CIV). When *Ngb*-treated mice were compared, it was observed an increase of 90%, 77.8%, or 97.8% in *Ngb*-treated cerebella for CI, CIII, or CIV relative to values measured in cerebella from *Ngb*-treated controls (*p* = 0.99, 0.34 or >0.999 for CI, CIII or CIV, respectively). Hence, *Aifm1* or *Ngb* overexpression was sufficient to rescue CI, CIII, and CIV deficiencies in *Hq* cerebella (Figure 7A, upper panel). It is worth mentioning that the observed improvements of respiratory chain CI, CIII, and CIV activities were not different when AAV2/9-*Aifm1* or AAV2/9-*Ngb* vector was used (*p* = 0.81, 0.91, and 0.92, respectively). Data collected for complex V activity in cerebellar homogenates were more heterogeneous, yet the differences were not significant when cerebellar homogenates from control and *Hq* mice subjected to gene therapy were compared (Figure 7A, bottom panel).

Next, enzymatic activities of citrate synthase (CS) and malate dehydrogenase (MDH), which are markers of mitochondrial integrity as described in brain from neonatal mice^49^^,^^50^ and as we observed in cerebella from 8-month-old untreated *Hq* mice relative to their age-matched counterparts were evaluated in cerebella from treated mice (Figure 7A, bottom panel).^39^ When the cerebella from *GFP*-treated *Hq* and *GFP*-treated controls were compared, it was observed a 36.9% diminution in CS activity in *Hq* mice. The CS activity increased by 64% and 61% in cerebella from *Hq* mice treated with AAV2/9-*Aifm1* or AAV2/9-*Ngb*, respectively, when compared with *GFP*-treated *Hq* mice (*p* = 0.0036 or 0.0038). The activities assessed in cerebella from *Hq* mice treated with AAV2/9-*Aifm1* or AAV2/9-*Ngb* reached the values measured in cerebella from control mice treated with the same vectors (*p* = 0.992 or *p* = 0.998, respectively). Moreover, no difference was observed between CS activity in cerebella from *Hq* mice treated with AAV2/9-*Aifm1* or AAV2/9-*Ngb (p* > 0.999).

We also observed a 28% decrease in the activity of MDA, the second enzyme of the TCA cycle assessed, in cerebella from *Hq* mice treated with AAV2/9-*GFP* relative to their control counterparts (*p* = 0.0042). The activity of MDH was increased by 32.3% or 29.2% for AAV2/9-*Aifm1* (*p* = 0.002) or AAV2/9-*Ngb* relative to *GFP*-treated *Hq* mice (*p* = 0.0016). The activities of MDH in cerebella that overexpressed *Aifm1* or *Ngb* were, as for CI, CIII, and CIV, very similar to those measured in their control counterparts; *p* = 0.977 or 0.974, respectively (Figure 7A, bottom panel).

To corroborate the benefit of gene therapy for mitochondrial function, we measured the amounts of ATP, ADP, and AMP using ultra-high performance liquid chromatography coupled with tandem mass spectrometry (UHPLC-MS/MS) (Figure 7B, upper panel). The amounts of three nucleotides were significantly diminished in the cerebella from 8-month-old *Hq* mice relative to 8-month-old controls. Cerebella from *Hq* mice treated with AAV2/9-*Aifm1* or AAV2/9-*Ngb* accumulated 80.8% (*p* = 0.085) and 72.6% (*p* = 0.15) more of AMP than untreated *Hq* mice. The increase of ADP amounts was of 202% (*p* > 0.999) and 215.3% (*p* = 0.043) in cerebella treated with AAV2/9-*Aifm1* or AAV2/9-*Ngb* when compared with untreated *Hq* mice, respectively. The amount of ATP in cerebella overexpressing *Aifm1* or *Ngb* was 4- or 7-fold higher relative to untreated *Hq* mice (*p* = 0.214 or 0.0014, respectively); apparently the beneficial impact of *Ngb* overexpression was better than the *Aifm1* one (Figure 7B, upper panel).

Next, the energy charge was determined since it represents a useful indicator for understanding the overall energy flow between energy-utilizing reactions and energy-generating reactions. It tends to the unity in tissues with maximum energy status and decreases to approach zero in tissues exhibiting respiratory chain deficiency.^51^^,^^52^ A rise in energy expenditure would result in a decrease of energy charge (EC), unless a concomitant increase in the rate of phosphorylation of ADP to ATP occurs, via a functional respiratory chain. Because of decreased nucleotide pools in cerebella from untreated *Hq* mice relative to those measured in treated *Hq* cerebella, EC was significantly different in AAV2/9-*Aifm1* or AAV2/9-*Ngb*-treated cerebella of *Hq* mice relative to untreated *Hq* mice. In cerebella from *Aifm1*- and *Ngb*-treated *Hq* mice, it is observed a 1.36- and 2.09-fold increase of EC relative to cerebella from untreated *Hq* mice; *p* = 0.21 and 0.0014 (Figure 7B, upper panel). Accordingly, the EC in cerebella from *Hq* mice treated with AAV2/9-*Ngb* was almost identical to the one measured in untreated 8-month-old control mice (*p* = 0.721). In view of these results, the positive impact of AAV2/9-*Aifm1* treatment seems inferior to the one observed with AAV2/9-*Ngb* for the content of ATP as well as for the EC value. This is why the difference, when the EC values were compared between *Aifm1*-treated and *Ngb*-treated cerebella, was significant (*p* = 0.0006).

Electrons can leak prematurely to oxygen from electron carriers associated with substrate catabolism and the respiratory chain; in physiological conditions if electrons are transferred efficiently to complex IV there is generation of H_2_O, which drive OXPHOS, though if they leak individually or in pairs, they generate superoxide radicals and hydrogen peroxide, respectively. To date, six sites localize in the complex I and five sites operate within the complex III. It is admitted that in physiological conditions, 0.15% of daily O_2_ consumption is converted into the superoxide anion, a precursor of hydrogen peroxide and hydroxyl free radicals in the brain. If the central nervous system exhibits, for any reason, low antioxidant defense mechanisms, it is sensitized to oxidative stress, which could result in cell death.^53^^,^^54^

Antioxidant mechanisms in the brain are composed of enzymes such as superoxide dismutase, catalase glutathione reductase, and glutathione peroxidase. Aminothiols (sulfur-containing amino acids) are biological compounds exhibiting numerous vital cellular functions; one of the most relevant is their role as antioxidant systems. The major cellular aminothiols are glutathione (GSH), cysteine, and homocysteine; they and their oxidized disulfide counterparts are carefully regulated to maintain redox homeostasis within the cell and so to protect from oxidative and xenobiotic stressors. So, reduced to oxidized ratios of GSH-GSSG, cysteine-cystine, and homocysteine-homocystine are suitable indicators of oxidative stress and cellular redox status.^55^^,^^56^ Thus, we performed UHPLC-MS/MS analyses to simultaneous determine reduced/oxidized aminothiols in cerebellar homogenates.

The Figure 7B (bottom panel) shows decreased values for GSH/GSSG, cysteine/cystine and homocysteine/homocystine in cerebella from 8-month-old untreated *Hq* mice relative to those of age-matched controls indicating oxidative damage: 70.5% reduction for GSH/GSSG (*p* = 0.0003), 95.6% reduction for cysteine/cystine (*p* < 0.0001) and 86.2% reduction for homocysteine/homocystine (*p* = 0.0002). On the contrary, for the three ratios measured, *Ngb* overexpression led to increased values relative to the ones measured in untreated *Hq* cerebella (395.4%, 776.0%, and 513.7%) and in AAV2/9-*Aifm1*-treated *Hq* cerebella (227.1%, 378.0%, and 255.6%). When the three ratios were compared between untreated *Hq* cerebella and *Hq* cerebella that overexpressed *Aifm1*, no significant differences were observed (*p* = 0.27, 0.58, and 0.25). On the contrary, *Ngb*-overexpressing cerebella exhibited significantly different ratios from untreated *Hq* mice (*p* = 0.0005, 0.0005, and 0.0011). Therefore, *Hq* mice exhibited severe oxidative stress in their cerebella at the age of 8 months; gene therapy counteracts this effect with NGB being much more efficient to protect against oxidative stress than AIF.

### Gene therapy preserves cognitive and motor performances of *Hq* mice

*Hq* mice aged 2 and 8 months had reduced responses in an array of cognitive and motor tests, relative to age-matched counterparts.^39^ To assess whether preventing mitochondrial functional failure and cerebellar degeneration of *Hq* mice through *Aifm1* or *Ngb* overexpression can result in better cognitive and motor abilities, we studied the behavioral responses of control and *Hq* mice following treatment with AAV2/9-*GFP* (negative control), AAV2/9-*Aifm1*, or AAV2/9-*Ngb*.

**Figure 8:**
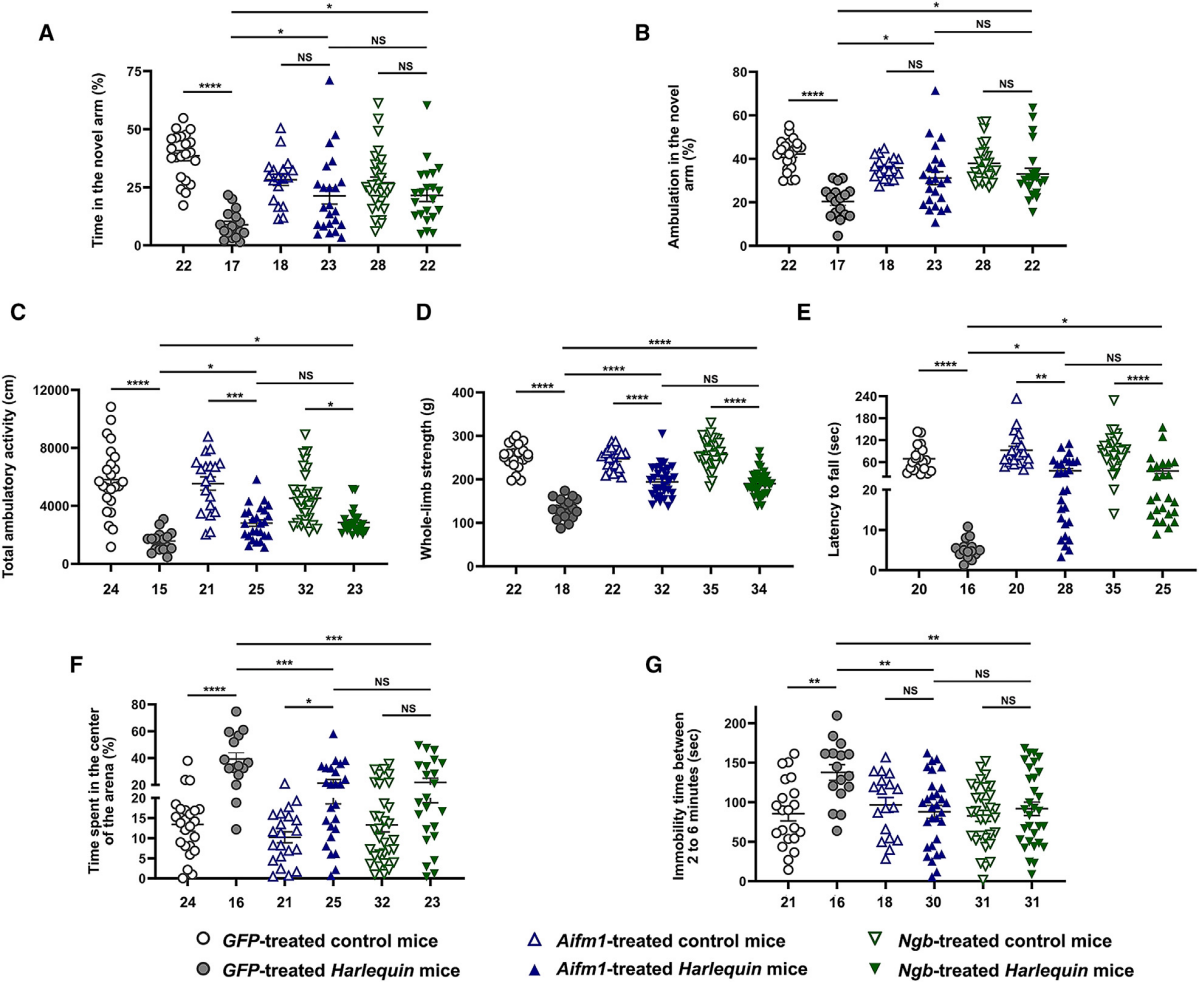
Assessment of motor and cognitive capacities of *Harlequin* mice subjected to gene therapy Control and *Hq* mice aged 2 months were subjected to gene therapy via stereotactic surgery, to deliver AAV2/9-*GFP*, AAV2/9-*Aifm1*, or AAV2/9-*Ngb* vector to each cerebellar hemisphere. Six months after vector administration, mice were subjected to an array of behavioral tests. (A and B) Representations of data obtained from two parameters evaluated with the Y-Maze test. (C) The overall locomotor activity of each mouse was evaluated by the measurement of the distance traveled in the whole arena (cm) of an open field device during the total duration of the test, i.e., 20 min. (D) Whole-limb grip strength test: Six trails were performed per mouse; this assay allows estimation of the muscular power of the four limbs and the mean ± SEM were plotted in the histogram shown. (E) Motor coordination and balance were assessed with the accelerating rotarod test, the latency time to fall in seconds is illustrated in the histogram as the mean ± SEM. (F) The open field test was used to estimate anxiogenic-like behavior. The percentage of time that the mouse remains close to the walls vs. in the center of the arena, during the first 10 min of the test, was plotted on the bar graph illustrated. (G) Stress-coping behavior was assessed by the tail suspension test, which allows measuring the immobilization time. This time is calculated during the last 4 min of the trail. The values for each test (A–G) were plotted using GraphPad Prism 10.2 software; they correspond to means ± SEMs. The number of animals evaluated per group is indicated below each bar chart. *p* values, from GraphPad Prism in each histogram are as follow: *p* > 0.05: NS (not significant); ∗*p* ≤ 0.05; ∗∗*p* ≤ 0.01; ∗∗∗*p* ≤ 0.001; ∗∗∗∗*p* ≤ 0.0001. The detailed statistical analyses of the data are available in Table S3.

For the Y-Maze test, *GFP*-treated *Hq* mice exhibited reduced spatial reference memory with the percentages of time spent and of distance traveled in the novel arm, which attained only 23.1% and 48.2% of the values measured in the *GFP*-treated control mice, respectively (*p* < 0.0001). Thus, *GFP*-treated *Hq* mice did not identify the novel arm as they have impaired recall of which arm was inaccessible in the first phase of the test. The lack of differentiation between the novel and other arms indicated their compromised memory (Figures 8A and 8B).

Conversely, when *Hq* mice treated with AAV2/9-*Aifm1* were compared with control mice treated with the same vector, their responses reached 75.4% and 86.7% for the time spent in the novel arm and the distance traveled inside it (*p* = 0.184 and 0.988, respectively; Figures 8A and 8B). *Hq* mice treated with AAV2/9-*Ngb* exhibit, equally, an improvement in their memory since the percentages reached 79.9% and 87.2% for the time spent and the distance traveled in the novel arm relative to control mice treated with the same vector (*p* = 0.89 and 0.61, respectively; Figures 8A and 8B). Moreover, overexpression of *Aifm1* or *Ngb* resulted in improved responses when compared with *Hq* mice treated with AAV2/9-*GFP* (Figures 8A and 8B). For the time spent in the novel arm, we observed a 2.39- and 2.42-fold increase, respectively (*p* = 0.029 or 0.026). Besides, for the distance traveled in it, we observed a 1.53- or 1.62-fold increase when compared with *GFP*-treated *Hq* mice (*p* = 0.032 or 0.017). Hence, gene therapy, independently of the vector used, led to the reestablishment of mice preferences for the novelty because their memory for previously encountered spaces remained efficient.

Treated *Hq* and control mice were also subjected to the open field test to estimate their overall locomotor activity expressed as the total distance covered (in cm) during the entire duration of the test (20 min). Figure 8C illustrates the comparison of the data collected in mice treated with either one of the vectors; locomotor abilities of control mice were very similar, independently of the vector administered. Conversely, total ambulatory distance covered in the maze by AAV2/9-*GFP*-treated *Hq* mice was significantly reduced (27.4%) relative to control mice treated with AAV2/9-*GFP* (*p* < 0.0001). The treatment of *Hq* mice with either AAV2/9-*Aifm1* or AAV2/9-*Ngb* resulted in a better ability of the mice to move in the maze, which attained 50.8% or 63.0% of the locomotor ability measured in control mice treated with the same vectors (*p* = 0.0004 or 0.0282; Figure 8C). The hypo-locomotion of *GFP*-treated *Hq* mice was exacerbated relative to *Hq* mice treated with AAV2/9-*Aifm1* or AAV2/9-*Ngb*, which exhibited, respectively, 1.79- or 1.80-fold improved locomotor ability (*p* = 0.058 or *p* = 0.049, respectively, for AAV2/9-*Aifm1* or AAV2/9-*Ngb*). The administration of either AAV2/9-*Aifm1* or AAV2/9-*Ngb* in the cerebella from *Hq* mice resulted in similar locomotor performances (*p* > 0.999).

The grip strength test was used to estimate the muscle strength of the four limbs in treated control and *Hq* mice (Figure 8D). *Hq* mice that received the AAV2/9-*GFP* vector were severely impaired regarding the strength they can deploy to grip the grid of the apparatus, since there was a 47.5% diminution relative to age-matched controls treated with the same vector (*p* < 0.0001). On the contrary, the administration of AAV2/9-*Aifm1* or AAV2/9-*Ngb* to *Hq* mice resulted in muscle strengths reaching 78.6% and 73.1% of values measured in wild-type counterparts treated with the same vectors. Muscle strength remained frail in *Aifm1*- or *Ngb*-treated *Hq* mice relative to their wild-type counterparts (*p* < 0.0001). However, it is worth mentioning that their performance was significantly different from the one observed in *GFP*-treated *Hq* mice with a 46.5% and 43.6% augmentation (*p* < 0.0001 for both vectors). This confirms the benefits on the muscle strength in *Hq* mice that overexpressed in their cerebella *Aifm1* or *Ngb*.

Next, motor coordination was assessed using the accelerating rotarod task; *Hq* mice treated with the AAV2/9-*GFP* vector displayed poor rotarod performance, which was estimated by the time, in seconds, to fall from the device. *Hq* mice treated with the AA2/9-*GFP* vector reached only 7.7% when compared with *GFP*-treated control mice (Figure 8E). On the other hand, *Hq* mice that overexpressed in their cerebella either *Aifm1* or *Ngb*, while underperforming in the task relative to their wild-type counterparts, exhibited an overall enhanced motor coordination when compared with *GFP*-treated *Hq* mice. Indeed, they reached, respectively, 39.2% and 40.6% of the values measured in control mice treated with either AAV2/9-*Aifm1* or AAV2/9-*Ngb*, though remaining statistically different (*p* = 0.0013 or <0.0001, respectively). Overexpression of *Aifm1* or *Ngb* resulted in a 6.88- or 6.77-fold increase in the time to fall relative to *GFP*-treated *Hq* mice (*p* = 0.012 or *p* = 0.018, respectively). These data indicate that coordination defects in *Hq* mice were less severe after gene therapy regardless of the vector used.

Open Field allowed estimation of the tendency of mice to remain at the edge of the box or close to its walls (thigmotaxis) during the first 10 min of the test (Figure 8F). It was noticed that *GFP*-treated *Hq* mice displayed reduced anxiety-like traits relative to *GFP*-treated control mice corresponding to a 2.93-fold increase in the time that *Hq* mice spent in the center of the arena relative to their control counterparts (*p* < 0.0001). Treatment with AAV2/9-*Aifm1* or AAV2/9-*Ngb* led a reduction of 54% or 55.8% in the time spent in the center of the arena relative to the one measured in *Hq* mice treated with AAV2/9-*GFP* (*p* = 0.0001 or 0.0002, respectively), confirming the benefit for these mice of *Aifm1* or *Ngb* overexpression in their cerebella. Moreover, the responses in *Hq* mice that overexpressed *Aifm1* or *Ngb* were not statistically different (*p* > 0.999).

Last, we subjected treated mice to the tail suspension test since it is considered as a reliable assessment of depressive-like phenotypes and is widely used for screening antidepressant drugs.^57^ In response to the stressful situation (mouse hanging by the tail), *GFP*-treated *Hq* mice exhibited an increase of 61% in the immobility time relative to *GFP*-treated controls indicating that *Hq* mice expressed a depressive-like behavior, *p* = 0.0005 (Figure 8G). In contrast, *Hq* mice treated with either AAV2/9-*Aifm1* or AAV2/9-*Ngb* responded to the test similarly than control mice treated with the same vectors (*p* = 0.58 or 0.53, respectively) and significantly different from *GFP*-treated *Hq* animals. Without a doubt, *Hq* mice in which cerebella overexpressed *Aifm1* or *Ngb* displayed a shortening of immobility extent relative to *GFP*-treated *Hq* mice; values attaining 36.1% or 33.3% of the value in *GFP*-treated *Hq* mice (*p* = 0.0094 or 0.0153, respectively). Thus, gene therapy allowed mice to adopt a stress-coping behavior similar to the one of control mice without significant difference between *Aifm1* and *Ngb* overexpression (*p* > 0.999).

Altogether these tests revealed that overexpressing *Aifm1* or *Ngb i*n the cerebellum of *Hq* mice efficiently mitigated or even prevented the deterioration of motor and cognitive functions.

## Discussion

The cerebellum is responsible for an array of motor, cognitive, and affective functions through numerous cerebellar-subcortical-cortical circuits.^58^ Cerebellar ataxia refers to the dysfunction of the cerebellum that leads to difficulties with gait and balance, eye movements, speech, linguistic communication, hand dexterity, learning and memory, spatial cognition, and behavior.^59^^,^^60^

Hereditary ataxias are divided, according to the transmission mode, into autosomal dominant cerebellar ataxias, also known as spinocerebellar ataxias (SCAs), autosomal recessive cerebellar ataxias (ARCAs), mitochondrial ataxias, and X-linked ataxias. Both SCAs and ARCAs are due to Purkinje cell damage with progressive cell loss in SCAs or altered synapses/impaired dendritic architecture in ARCAs.^61^^,^^62^^,^^63^

Purkinje cells are one of the largest neurons of the brain and the sole neurons sending outputs from the cerebellum; they receive more excitatory synaptic inputs than any other neuron in the brain. It is estimated that a human PC has about 200,000 dendritic spines, their axons travel into the DCN, where they make about 1,000 synapses with several types of neurons.^64^^,^^65^

The high metabolic activity, large size, and expansive dendritic arbor of PCs result in their vulnerability to changes in tissue environment or genetic mutations.^66^ Reduced energy supply/mitochondrial dynamics disruption/altered intracellular calcium homeostasis in cell somas, dendritic arbors, or axons constitute frequent causes underlying PC loss or dysfunction in cerebellar ataxias.^66^^,^^67^^,^^68^

Almost half of patients suffering from primary mitochondrial diseases (PMDs) display ataxia as the most common movement disorder at onset, which is related to the degeneration of Purkinje cells.^63^

Currently, the handling of hereditary ataxias is limited to genetic counseling and symptomatic management (e.g., motor and speech rehabilitation programs or noninvasive cerebellar stimulation). But as yet no neuroprotective strategies exist to durably reduce the deleterious progression of the pathology and thereby to improve a patient’s daily life.^59^^,^^69^

The aim of our study was to implement, via gene therapy, a treatment that effectively and permanently preserves mitochondrial homeostasis of cerebellar neurons regardless the mutation responsible for tissue degeneration. The gene therapy candidate chosen is the neuroglobin (NGB), a highly conserved member of the globin family in mammals, discovered in 2000.^25^ Biochemical and biophysical characterization of the NGB allows to assign physiologically relevant activities to the protein such as electron transfer, oxygen supply, and protection against oxidative stress.^29^ Up-to-date an array of hypotheses about its function is under intense debate but its neuroprotective role has been largely demonstrated *in vitro* and *in vivo*.^28^^,^^29^^,^^70^^,^^71^ Furthermore, it is now well recognized that the majority of NGB localizes to the mitochondria, as we described in rodent retinas, in which we showed that NGB is required for respiratory chain function.^30^^,^^31^ Consequently, researchers worldwide are considering NGB upregulation or even its targeted delivery for treating neurological disorders given the concurring evidence of NGB induction upon several pathologies or cellular insults.^72^

Our previous studies have demonstrated NGB capacity to protect visual function in *Hq* mice, which is a model of primary mitochondrial disease.^31^^,^^32^ To better understand the involvement of NGB in neuroprotection, we targeted the cerebellum from these mice since they develop ataxia as they age.^33^^,^^35^ Moreover, *Hq* mice are considered as a relevant model of recessive X-linked mitochondrial disease due to *AIFM1* mutations because of the nearly complete absence of the mitochondrial protein AIF^33^ in all their tissues. AIF regulates OXPHOS and energy homeostasis by assisting biogenesis and/or stabilization of respiratory chain complexes.^73^

Patients harboring deleterious *AIFM1* mutations had severe neurological impairments in addition to axonal polyneuropathy.^74^ Interestingly, cerebellar ataxia is considered a prominent hallmark of these patients.^75^^,^^76^^,^^77^

Morphological and functional evaluations of the cerebellum from *Hq* mice allowed us to identify several features of the pathogenic process leading to ataxia in adult mice. In our hands, *Hq* mice exhibit cerebellar damage as early as 2 months of age, with aggravation of the phenotype 6 months later.^39^

Accordingly, we designed a gene therapy protocol using recombinant AAV2/9 vectors that allow the overexpression of murine *Ngb* or *Aifm1* to provide the proof-of-concept that NGB is able to functionally compensate AIF absence in the cerebellum of *Hq* mice. Recombinant AAV2/9 vectors harboring *Ngb* or *Aifm1* coding sequence were directly delivered to cerebellar hemispheres via stereotactic surgery in 2-month-old mice. Transduction yield in Purkinje cells was 54.7% in *Hq* mice and 73.6% in control mice (Figure 2); this difference could be due to both the age of mice when they were subjected to gene therapy and the time required for reaching the peak of protein synthesis from the single-stranded DNA vectors used. The use of AAVs with single-stranded genomes delays for about 4 weeks the accumulation of proteins synthesized from the transgenes, as single-stranded AAV genomes need to be converted into double-stranded DNA, a rate-limiting step during AAV transduction.^41^ So, the treatment of *Hq* mice at the age of 2 months could be too late for efficiently preventing neuronal cell loss. Despite the delayed accumulation of AIF and NGB in *Hq* mice, the abundance of AIF and NGB were 5.4-fold and 2.7-fold more significant in treated *Hq* cerebella than their untreated counterparts, 6 months post-injection without changing the intracellular localization of the proteins, mostly inside the mitochondria (Figure 4). Consequently, we observed a less noticeable decrease of cerebellar weight and surface, the survival of numerous Purkinje cells, the preservation of their dendritic arbor complexity, as well as a significant increase in the density of synapses between their axons and neurons in the DCN (Figures 2, 3, 4, 5, and 6). For instance, the number of Purkinje cells in the posterior region of the tissue displayed an increase of 68.2% and 81.3% in *Aifm1*-treated *Hq* mice and *Ngb*-treated-*Hq* mice relative to *GFP-*treated *Hq* mice (Figure 3C). Furthermore, the remaining Purkinje cells in treated *Hq* mice exhibited extended and elaborated dendrites within the molecular layer; hence its thickness in lobule IX of cerebella from *Hq* mice treated with AAV2/9-*Aifm1* or AAV2/9-*Ngb* was increased by 64.5% or 64.4% relative to AAV2/9-*GFP-*treated *Hq* mice (Figure S3).

Along with the preservation of cerebellar morphology, we demonstrated the significant protection of mitochondrial bioenergetics status, by measuring enzymatic activities of respiratory chains CI, CIII, and CIV, CS, and MDH as well as nucleotide pools and EC (Figure 7A). Undeniably, we observed an almost full restoration of the three enzymatic activities assessed in *Hq* cerebella that overexpressed *Aifm1* or *NGB* with no difference between the two genes. Moreover, CS and MDH activities were also enhanced in *Hq* mice treated with AAV2/9-*Aifm1* or AAV2/9-*Ngb* relative to AAV2/9-*GFP-*treated *Hq* mice. Mitochondrial vulnerability was recently evidenced in brain from rats subjected to neonatal hypoxia-ischemia due to significant decreased activities of the TCA cycle enzymes, CS and MDH.^50^ Therefore, we show here that gene therapy, not only improved respiratory chain activity but was beneficial for the overall organelle homeostasis (Figure 7A). To corroborate the benefit of gene therapy for mitochondrial OXPHOS, UHPLC-MS/MS analyses were performed to determine the amounts of ATP, ADP, and AMP since they are indicative of cerebellar metabolic status. These values allowed the calculation of the EC, a quantitative measure of the balance between adenine nucleotides^51^ (Figure 7B). Interestingly, the amount of ATP in cerebella overexpressing *Aifm1* or *Ngb* was 4- or 7-fold higher relative to untreated *Hq* mice; apparently the beneficial impact of *Ngb* overexpression was better than the *Aifm1* one. Consequently, a difference between *Ngb* and *Aifm1* overexpression in cerebella from *Hq* mice was evidenced when the EC was calculated (Figure 7B). Indeed *Aifm1*-treated *Hq* and *NGB*-treated *Hq* cerebella were significantly different (*p* = 0.0006). The positive impact of AAV2/9-*Aifm1* treatment was inferior to the one observed with AAV2/9-*Ngb* for ATP content and EC value. Using UHPLC-MS/MS, we demonstrated an additional difference between AIF and NGB mode of action; indeed, oxidative stress and cellular redox status indicators values of GSH/GSSG, cysteine/cystine and homocysteine/homocystine were increased in AAV2/9-*Ngb*-treated cerebella relative to the ones measured in AAV2/9-*Aifm1*-treated ones (Figure 7B). Therefore, the increased abundance of NGB, as for ATP pools and EC, was much more efficient to protect against oxidative stress than the one of AIF. Therefore, it is likely that *Ngb* overexpression preserves better the bioenergetic status of *Hq* cerebella than *Aifm1* overexpression.

TEM confirmed the valuable benefit of *Aifm1* or *Ngb* overexpression for the preservation of mitochondrial morphology within PCs and of their axons, which established more connections with neurons in the DCN (Figure 6). Elevated enzymatic activities of respiratory chain and TCA cycle (Figure 7) should be involved in the preservation of mitochondrial morphology.

The conservation of cerebellar morphology and mitochondrial function due to *Ngb* or *Aifm1* overexpression leads to an improvement of motor and cognitive performances in treated *Hq* mice: muscular strength, stress-coping behavior, motor coordination, locomotor activity, inherent preference for the novelty, and spatial reference memory (Figure 8). Spatial reference memory, corresponding to higher-order cognitive processing, has been assigned to the cerebellum,^47^^,^^78^ and also indicates impaired functioning of the hippocampus. The functional connectivity between the hippocampus and the cerebellum, including crus1 and lobules VI, IV/V, IX, and X has been evidenced in mice subjected to navigation tasks. Thus, the hippocampal-dependent memory is functionally associated with cerebellum activity.^79^^,^^80^ Spatial reference memory is reduced in *Hq* mice suggesting that the hippocampo-cerebellar network is impaired; indeed *GFP*-treated *Hq* mice attained only 23.1% of the value measured in the *GFP*-treated control mice for the time spent in the novel arm of the Y-Maze; such as these mice did not differentiate the novel arm from the two other arms signified that they displayed compromised memory (Figure 8). Overexpression of *Aifm1* or *Ngb* resulted in improved responses when compared with *Hq* mice treated with AAV2/9-*GFP*; thus, a 2.4-fold increase was observed for the time spent in the novel arm (Figure 8). Hence, gene therapy, independently of the vector used, led to the reestablishment of the inherent preference of mice for the novelty by revealing efficient memory for spaces tested in the initial phases of the test.

We also assessed two parameters in *Hq* mice after gene therapy that are relevant of anxiety-like behavior, and depression-related behavior by subjecting mice to open field and tail suspension tests (Figure 8). These behaviors are generally considered as hippocampus/amygdala dysfunctions and recently it has been shown the link between the cerebellum and these regions in mice.^80^^,^^81^

Two main conclusions can be raised: (1) the difference between times spent in the center of the open field arena was significant between *Aifm1*- or *Ngb*-treated *Hq* mice and *GFP*-treated *Hq* mice (56.4% or 58.1% reduction) confirming the benefit of *Aifm1* or *Ngb* overexpression; (2) *Hq* mice in which cerebella overexpressed *Aifm1* or *Ngb* displayed a shortening of immobility time relative to *GFP*-treated *Hq* mice; values attaining 36.1% or 33.3% of the value in *GFP*-treated *Hq* mice. These mice exhibited a reduction in depression-related behavior assessed with the tail suspension test; the response was similar to the one of control mice without significant difference between *Aifm1* and *Ngb* overexpression (Figure 8). Therefore, gene therapy was beneficial for restoring normal anxiety-like and depression-related behaviors in *Hq* mice.

Noteworthy, reduced anxiety in the open field test has been demonstrated in *mGluR5* (metabotropic glutamate receptor 5) knockout mice.^82^ Excessive mGluR5 signaling has been proposed as one of the causal factors for multiple neurodevelopmental disorders.^83^ In 2011, it was also described that a cerebral *Erk2* knockout mouse exhibited marked deficits in long-term memory and decreased anxiety-related behaviors^84^ as those we described here for the *Hq* mice. Erk2, as mGluR5, might be another factor underlying human psychiatric disorders; interestingly, patients harboring deleterious *AIFM1* mutations often suffer from mental retardation or intellectual disability.^85^^,^^86^

Regarding the overall benefits of gene therapy for cerebellar integrity in *Hq* mice, two remarks can be made: (1) the positive consequences of *Aifm1* or *Ngb* overexpression for cerebellar neurons were almost identical for all the parameters evaluated in *Hq* mice 6 months post-injection except for ATP content, EC, and oxidative stress markers, which were better preserved in *Ngb*-treated *Hq* mice relative to *Aifm1*-treated *Hq* mice (Figure 7B); and (2) despite these beneficial changes, cerebellar morphology and function ameliorations were incomplete and resulted in a partial, while significant, diminution of the ataxic phenotype in *Hq* mice.

In our previous study of blindness prevention in *Hq* mice via *Ngb* overexpression, we observed a comparable efficacity of both AAV2/2-*Ngb* and AAV2/2-*Aifm1* vectors for the protection of visual function. Thus, despite that no overlapping function between AIF and NGB has been identified, *Ngb*-mediated gene therapy was sufficient to bypass the absence of AIF in retinas from *Hq* mice by preventing the bioenergetics crisis in retinal ganglion cells, thereby resulting in a better visual function.^31^ The present study also demonstrated an equivalent efficacy of *Ngb* and *Aifm1* overexpression for preserving the functionality and the viability of Purkinje cells in *Hq* mice.

Moreover, to seek a better therapeutic response, the treatment can be performed in animals aged 1 month instead of 2 months via intravenous injection in the retro-orbital sinus, which represents a systemic delivery of gene therapy vectors, widely accepted as an ideal and safe route for venous injection that in *Hq* mice will be less damaging for the cerebellum.^87^ Besides, self-complementary AAV (scAAV) genomes can be used to bypass the limiting step of the second strand DNA synthesis.^41^

Ultimately, the present study makes the proof-of-concept that *Ngb*-mediated gene therapy lastingly preserves mitochondrial robustness in cerebellar neurons. Therefore, *Ngb* overexpression represents a pioneering tool to ameliorate life conditions of patients suffering from cerebellar ataxias.^88^

## Materials and methods

### Mice

The *Harlequin* (*Hq*) mice originate from the *C57BL/6J* strain *B6CBACaA*^*w-*J^/A-Pdc8^*Hq*^/J; they were shipped from The Jackson Laboratory (http://jaxmice.jax.org/strain/000501.html). All hemizygous (*Hq*/Y) male mice used in this study were between F2 and F5 mice bred from founders (*Hq*/X female mice with wild-type male mice). Only the hemizygous (*Hq*/Y) male mice and their male littermates were evaluated and subjected to gene therapy. The mice were housed in a pathogen-free barrier facility with one to four animals per cage in a temperature-controlled environment, with a 12-h light/dark cycle and free access to food and water. The animal facility (PHENO-ICMice) is located at the Paris Brain Institute (https://institutducerveau-icm.org/fr/pheno-icmice-rodent-preclinical-phenotyping-service/).

Animal care and all experimental procedures were conducted in accordance with European Community Council Directive 2010/63/UR, on the protection of animals used for scientific purposes. The scientific project has been authorized regarding the rules on the care and use of animals in research by the internal scientific committee of the Brain Institute (P128R) and the French Ministry of Research (2410). It was also approved by the ethics commission of Sorbonne University (2017032721505008).

### Production of single-stranded DNA AAV vectors

AAV serotype 9 AAV2/9-*Aifm1* and AAV2/9-*Ngb* vectors have been obtained as for the serotype 2.^31^^,^^32^ Briefly, murine sequences were cloned into the pAAV-IRES*-hrGFP* vector (Agilent Technologies). The AAV2/9-*Ngb* vector contains the open reading frame, ORF (453 base pairs [bp]), the 5′ untranslated region (UTR) (279 bp), and the 3′ UTR (895 bp) of the mouse *Ngb* mRNA variant 2 (NCBI: NM_022414.2). The AAV2/9-*Aifm1* vector contains the 5′ UTR (87 bp), the entire ORF (1836 bp), and the 176-bp full-length 3′ UTR of the mouse *Aifm1* mRNA (NCBI: NM_012019). The presence in each construction of the full UTR sequences guarantees mRNA stability and translation capacity.^89^^,^^90^ The expression cassettes flanked by the two inverted terminal repeats (ITRs) were packaged in AAV9 shells to ensure a high yield of neuronal cell transduction.^91^^,^^92^ Additionally, each plasmid possesses a cassette allowing the expression of the recombinant humanized green fluorescent protein (hrGFP) translated from the encephalomyocarditis virus internal ribosome entry site. This feature allows determination of the transduction yield of Purkinje cells using cerebellar sections subjected to immunochemistry with antibodies against GFP and calbindin, a reliable marker of Purkinje cells.^40^ The physical map of each construct is illustrated in Figure S1.

Vectors were produced by the Translational Vector Core of the INSERM UMR1089 research unit at Nantes, France (http://umr1089.univ-nantes.fr).

### Stereotactic surgery

The day of the surgery, the mice underwent a volatile anesthesia (isoflurane 3% for induction and then 2.5% in a mask). Each mouse is placed in a stereotactic frame and maintained on a heating mat. An incision is made in the skin of the skull and two small-diameter holes are made with a mini-drill (carbon steel burrs 0.5 mm diameter) in the cranial bone on each side of the cerebellum. The coordinates (6 to 6.5 mm caudal from bregma and 1.75 to 2 mm laterally from the median line) were identified with the Atlas of Franklin and Paxinos (http://atlas.brain-map.org/) and correspond to each hemisphere of the tissue. The final concentration of each vector was 3 × 10^9^ vector genomes/μL; 2 μL are injected, i.e., 6 × 10^9^ vector genomes per cerebellar hemisphere with a depth of 1.1 mm ventral from the dura mater. The infusion rate is 0.35 μL/min; the vector is administered with a 10 μL-Hamilton neuro-syringe and a 33-gauge needle. To minimize the reflux of the vector solution, the neuro-syringe remained in place for 3 min, and then it was moved up by 0.3 mm every 45 s before its complete extraction.

Next, the surgical site was closed aseptically with absorbable surgical sutures. Immediately after the procedure, the mouse received subcutaneously an analgesic (buprenorphine 0.05–0.1 mg/kg) and 400 μL of physiological serum. The analgesic treatment was given every 8–12 h for 3–4 days after the surgery.

Stereotactic surgery is classified as moderate severity; thus, all postoperative care is given to minimize suffering after the intervention and to preserve "animal welfare."

During the course of this project, 139 *Hq* mice were subjected to gene therapy: 47 received AAV2/9-*Aifm1*, 55 received AAV2/9-*Ngb*, and 37 received AAV2/9-*GFP.* Additionally, 96 control mice were treated with either one of the vectors: 24 received AAV2/9-*Aifm1*, 46 received AAV2/9-*Ngb*, and 26 received AAV2/9-*GFP*. The protocol was particularly deleterious for *Hq* mice injected with AAV2/9-*GFP* for which 35.14% of injected animals died before the end of the experiment while injections with AAV2/9-*Aifm1* or AAV2/9-*Ngb* led to a loss of 23.40% or 23.64%. Overall, the following number of treated mice survived and were evaluated for their behavioral and cerebellar features: 36 *Aifm1*-treated *Hq*, 42 *Ngb*-treated *Hq*, 23 *GFP*-treated *Hq*, 23 *Aifm1*-treated controls, 45 *Ngb*-treated controls, and 25 *GFP*-treated controls.

Six months after vector administration, mice were euthanized, cerebella from about 60% of mice were reserved for studies of biochemistry/molecular biology, and the remaining 40% were used for histology or TEM evaluations.

### Behavior evaluations of mice

Treated control and *Hq* mice were evaluated to appreciate their cognitive and motor capacities 3 and 6 months after gene therapy. All the mice were also evaluated at the age of 7–8 weeks just before the administration of AAV2/9 vectors. The mice engaged in this study are housed at the Pheno-ICMice platform; thus, administration of the vectors and behavioral evaluations were performed at this facility. Because of their mixed genetic background, *Hq* mutant mice show variable phenotypic traits with obvious inter-individual differences, which has also been largely described in patients suffering from mitochondrial diseases.^14^ To obtain consistent and reproducible results, because of this heterogeneity, we performed experiments in large-sized animal cohorts as we described in our previous study.^39^ Besides, as mentioned in the previous section, an additional pitfall was encountered because of surgery severity that resulted in a diminished survival rate for *GFP-*treated *Hq* mice. Therefore, to obtain consistent results for morphological and functional evaluations of *GFP*-treated *Hq* mice, we needed to perform additional gene therapy experiments that encompassed also mice treated with AAV2/9-*Aifm1* or AVV2/9-*Ngb* to be evaluated at the same time for their behavioral characteristics. All of these experiments required us to be respectful of the rules on the care and use of animals in research and of the 3R guiding principles (Replacement, Reduction, and Refinement). Therefore, as soon as we received relevant data for the *GFP*-treated *Hq* group, we did not continue gene therapy experiments but analyzed for the behavioral features of all the treated mice. At the end, the overall number of analyzed mice differed between each group. Finally, for some tests a few mice had to be excluded as described in the literature, particularly for the assessment of cognitive skills.^93^^,^^94^ The experimental procedures were described in our previous study;^39^ the most important steps of each test are summarized below.

### Grip strength test

Muscle strength of four limbs per mouse was measured five to six times. The mean of the best recorded values (minimum of three) was calculated for each animal for data analysis.^95^

### Accelerating rotarod

We evaluated motor coordination and balance during 3 consecutive days. Mice were tested three times each day with a 15-min interval between each trial. The speed at the beginning of the test was 4 rpm and increased gradually for the following 5 min until 40 rpm.^96^ The speed and the latency time to fall from the device were recorded; the mean was calculated using the neighboring values of the nine trials for each animal.

### Open field

The mouse is placed in the middle of the apparatus (a square of 50 cm × 50 cm × 30 cm with a white Plexiglas floor and gray Plexiglas walls) and allowed to explore freely for 20 min; the behavior is recorded with an aerial tracking video camera connected to a computer. Two parameters were calculated: the total ambulatory activity and the time spent in the central zone of the device during the first 10 min of the test. The mouse’s preference to stay close to the walls of the arena or travel in the periphery is considered as an index of “anxiety-like” state, known as “thigmotaxis” and is based on the assumption that the center of the device is more threatening for rodents than the periphery.^97^ This behavior does not indicate anxiety per se but rather reflects the response of the mouse to two opposite options: explore a novel environment or avoid and escape from it.^97^^,^^98^

### Y-maze memory (forced alternation)

This test evaluates short-term spatial memory that requires interaction across different regions of the brain, such as the hippocampus and prefrontal cortex.^99^ During the first phase of the test, the animal is positioned in the starting arm and allowed to explore freely the apparatus for 10 min; in this phase one arm is blocked. After a 30-min rest, the animal is placed in the starting arm and is left to explore freely the apparatus, all arms open, for 7 min. Recorded videos and the Any-maze software allow calculation of the ratio between the time spent in the novel arm and the total time spent in the three arms during the second phase. The locomotor activity in the whole maze and within the novel arm are also calculated.^99^ Animals with impaired spatial reference memory will not be able to identify the novel arm, as they have insufficient recall of which arm was blocked in the initial phase of the test, leading to a lack of differentiation between the novel arm and other arms.^99^

### Tail suspension test

The tail suspension test consists of estimating the quantity of movements generated by the mouse while trying to escape from its suspension by the tail for the last 4 min of the test, which lasts 6 min. The immobility correlates with a “depressive-like behavior” since the animal does not have any possibility of escape or grip onto any surface.^57^^,^^100^

### TEM analyses

Ten treated *Hq* mice (five with AAV2/9-*Ngb* and five with AAV2/9-*Aifm1*), five control mice treated with AAV2/9-*NGB*, two untreated control mice, and four untreated *Hq* mice were employed for ultrastructural analyses using transmission electron microscopy (TEM).

Mice were anesthetized with a mixture of ketamine (140 mg/kg) and xylazine (8 mg/kg) by intraperitoneal injection and infused transcardiacally for 12 min with the fixative solution composed of 1% paraformaldehyde and 2.5% glutaraldehyde in 0.1 M cacodylate buffer at pH 7.4. The cerebella were removed and stored overnight at 4°C in the same solution. Cerebellar fragments (0.3 × 1 × 1 mm^3^) were post-fixed in 0.1 M cacodylate buffer pH 7.4 + 1% OsO_4_ for 1 h at 4°C and then in 1% aqueous uranyl acetate for 2 h at room temperature.

The samples were dehydrated in an ascending series of ethanol (30%, 50%, 70%, 95%, and three times 100%, 15 min each), then in ethanol/propylene oxide (1/1, v/v, 10 min), and eventually in pure propylene oxide (two times 10 min), before embedding in Epon.

Ultra-thin sections (80 nm) were prepared, stained with lead citrate, and photographed on a Jeol 100S transmission electron microscope (Jeol, Croissy-sur-Seine, France) equipped with a 2 × 2 k Orius 830 CCD camera (Roper Scientific, Evry, France).

#### Histologic evaluations

The mice were deeply anesthetized by intraperitoneal administration of Euthasol VET (0.35 mL/kg) and perfused through the ascending aorta with 10 mL of cold NaCl 0.9% and then with 100 mL of ice-cold fixative containing 4% paraformaldehyde in 0.1M phosphate buffer (PB, pH 7.4). After perfusion, the cerebellum was removed and post-fixed 3 h in PB + 4% paraformaldehyde and then cryoprotected in PB containing 30% sucrose for 2 days. The cerebellum was cut and frozen in cold 2-methyl-butan (at −50°C), and then kept at −80°C.

Floating sagittal sections of the cerebellum were obtained at a thickness of 40 μm with a cryomicrotome (Leica Biosystem SM2010R Sliding microtome). Sections were placed in a solution of PBS + sodium azide 0.03% in a 48-well plate (five sections per well). For long-term preservation at −20°C, the sections were kept in a solution for cryoconservation (PBS 0.5X + 20% ethylene glycol +30% sucrose).

For immunohistochemistry, the sections were washed twice for 10 min in PBS 1X + sodium azide 0.03%. Then, they were incubated in the blocking buffer (PBS 1X + sodium azide 0.03% + normal goat serum 10% + Triton X-100 0.3% + BSA 3%) for 2 h at room temperature with gentle stirring. Primary antibodies were added to the blocking buffer, at the appropriate concentrations, cerebellum sections with the antibodies remained overnight at 4°C with gentle stirring. Next day, cerebella were washed three times with PBS 1X + sodium azide 0.03% (10 min/wash) at room temperature and incubated in the blocking solution with secondary antibodies and DAPI for 2 h at room temperature in the dark with gentle stirring. After this incubation, cerebellar sections were rinsed three times with PBS 1X + sodium azide 0.03% (10 min/wash), and then mounted on glass slides.

The concentrations of the primary and secondary antibodies used for immunohistochemistry are shown in Table S1.

### Confocal microscopy and image analyses

Fluorescent labeling was visualized with a confocal laser scanning microscope (Leica TCS SP8); images were acquired with LAS X software and taken with the 20× objective or the 63× objective without zoom or applying a zoom factor of 0.75 (20× objective) or 2.5 (63× objective). Cerebellar sections were also scanned at 40× magnification with the digital whole-slide NanoZoomer Digital Pathology 2.0 HT scanner (Hamamatsu Photonics), using the Fluorescence Unit option (L11600-05) and the NanoZoomer’s 3-CCD TDI camera to estimate the number of Purkinje cells.

### Cell number counts

To count the number of Purkinje cells, the NDP viewer software was used with the reconstructed digital images of cerebellar sections belonging to the median region of the tissue (vermis) labeled with the antibody against the calcium binding protein Calbindin D-28k, a reliable marker of Purkinje cells.^40^ For each mouse, all the Purkinje cells (evidenced as calbindin-positive cells) within the vermis and the posterior lobe were manually counted: lobules VI to IX and lobule X (inferior vermis and also part of the flocculonodular lobe). The software allowed also to estimate the perimeter in millimeters of the zone counted within the Purkinje cell layer (PL). Hence, it is possible to calculate the Purkinje cell number per millimeter for this region of the tissue.

We used the same method to determinate the vector’s transduction yield by counting Calbindin-positive cells in lobules VI to X, which were also labeled with the antibody against the GFP.

The digital images used for cell number estimations were obtained for two to three independent immunohistochemical experiments. Additionally, cell numbers were estimated by three experimenters who were blinded to the administered vectors.

### Measurements of fluorescent signal intensities and cerebellar areas

ImageJ Fiji-win64 software (http://imagej.nih.gov/ij) was used to determine (1) the intensity of NGB or AIF staining in cerebellar sections; and (2) their overall area in mm^2^ for NGB- or AIF-labeled slides. Reconstructed cerebellar sections were generated by the NDP 2.0 HT scanner and exported as tiff images. With the ImageJ software, binary images were created for four to six sections per mouse; the scale for images was 1,097 pixels per mm. The thresholding was obtained with a dark background and by applying the Huang algorithm which allows delimiting and calculating automatically the total area (mm^2^).

To measure the molecular layer thickness, we used reconstructed sections for six cerebellar sections from mice treated with either *GFP*, *Aifm1,* or *Ngb* vectors; nine cerebellar sections from untreated control mice; and 10 cerebellar sections from untreated *Hq* mice. All the mice were euthanized at about 8 months of age. Cerebellar sections were selected from two independent labeling with anti-calbindin and β3-Tubulin antibodies. The distal region of lobule IX was chosen from each of them to obtain linear measurements (μm), from six segments drawn with the ruler tool of the NDP view software between the most exterior part of the lobule (molecular layer boundary) and the Purkinje cells layer.

It should be mentioned that acquisition parameters for the confocal microscope and the digital whole-slide scanner with respect to resolution, intensity, and thickness of synthetic focus images were kept as homogeneous as possible between the specimens from untreated control *Hq* mice as well as their treated counterparts.

### RNA extraction and real-time quantitative polymerase chain reaction

Half of cerebella were frozen and conserved at −80°C until use. Total RNAs were purified using the RNeasy plus mini kit from Qiagen; an additional DNAse step was added and then RNAs were cleaned using the RNeasy minElute cleanup from Qiagen. RNA concentration was measured with the Nanodrop device (ThermoScientific). One microgram of RNA per sample was reverse transcribed using the iScript cDNA synthesis kit containing oligo (dT) and random primers (Bio-Rad). The PCR reactions were performed with 10 ng of the reverse transcription product, a specific couple of primers per gene evaluated (Table S2) and the Sybr-green Master Mix (Bio-Rad) and run in the CFX384 (Bio-Rad). Biological samples were assessed in triplicate per gene. Ct values were obtained with the Bio-Rad CFX Maestro software. The *Rpl13a2* gene was used as reference to estimate the steady-state levels of *Aifm1*, *Ngb*, *Calbindin* (*Calb*), and *Nd4* (transcribed from mtDNA) mRNAs using the comparative ΔΔCt method.

### Western blot analysis

Proteins from the cerebellum were purified from eight control and eight *Hq* mice aged 8 months that were untreated, six *Hq* mice treated with AAV2/9-*Ngb*, and six *Hq* mice treated with AAV2/9-*Aifm1* at the age of 2 months and euthanized 6 months later. Half of each cerebellum was homogenized with 15x tissue weight/volume of the extraction buffer (TGNt): 50 mM Tris pH 7.4, 100 mM NaCl, 10% glycerol, 1% Triton X-100 + 1/100 of protease inhibitor cocktail (Thermo-Fischer Scientific) at 4°C with a 2 mL hand-driven glass-glass Potter-Elvehjem tissue grinder. Large cellular debris was discarded after centrifugation (1,000 × *g* for 5 min at 4°C). Protein amount in the supernatants was determined with the Bradford assay reagent (Sigma-Aldrich). Thirty micrograms of each sample were subjected to western blot analysis as described previously.^31^^,^^32^ Primary antibodies against NGB, AIF, TOMM 20, or β-Actin proteins and secondary antibodies were used at appropriate concentrations (Table S1). The antibody against NGB revealed three to four bands of approximately 25, 21, 19, and 17 kDa; the most consistent signal was the one corresponding to the 17-kDa signal. Thus, this signal was considered as the definite neuroglobin signal that was subsequently normalized against the β-actin signal. Specific bands were then detected with Clarity ECL Western Blotting Substrates (Bio-Rad). The apparent molecular mass of each protein was estimated by comparing each signal on the blots to the PageRuler Plus Prestained Protein Ladder (Thermo-Fischer Scientific) or SeeBlue Plus2 prestained Protein Standard (Thermo-Fischer Scientific). Signals from the membranes were visualized with the G:BOX Chemi and PXi imaging systems (Syngene, Cambridge, UK) then analyzed with GeneSys software (Syngene).

ReBlot Plus Strong Solution (Millipore, Molsheim, France) was applied to each PVDF membrane, enabling it to be used with three different antibodies consecutively.

Relative quantifications for different membranes were acquired by using the Quantity One analysis software (Bio-Rad, Marnes-la-Coquette, France). Protein steady-state levels were expressed as the ratio of each protein signal relative to the β-actin signal. The normalizations for 8-month-old untreated mice have been already shown in our manuscript currently under review; all the samples were run in parallel with those from treated mice.

### Tissue homogenate preparation and respiratory chain enzymatic assays

The half of each cerebellum was homogenized at 4°C in 800 μL for control mice or 600 μL for *Hq* mice of extraction buffer (250 mM sucrose, 20 mM Tris, 40 mM KCl, 2 mM EGTA, pH 7.2; BSA [fatty acid free] was added just before use to a final concentration of 1 mg/mL). The cerebellum solution was poured into a hand-driven glass-glass Potter-Elvehjem tissue grinder, and the tissue was broken up by 30 passages within the pestle of the homogenizer. Next, the homogenates were centrifuged at low speed (1,000 × *g* for 8 min at 4°C). The collected supernatants were subjected to two cycles of flash-freezing in dry ice, and the samples were stored at −80°C until use. Respiratory chain enzymatic activities were measured using a Cary 60 UV-Vis spectrophotometer (Agilent Technologies); the plots were collected using the Cary 60 Remote Diffuse Reflectance Accessory and the Cary WinUV Scan application. All the traces were baseline corrected and run over the range 360–830 nm.

Three spectrophotometric assays were carried out to measure sequentially the activity of respiratory chain complexes I-V, III, and IV in cerebellar homogenates as we performed in mouse retinas^31^^,^^32^ following the protocols previously published.^101^^,^^102^ The first assay measured the activity of complex I (CI: NADH decylubiquinone reductase), which is inhibited by rotenone.^101^ The experiments were performed at 37°C in triplicate with 35 μL of each cerebellar homogenate. The enzymatic activity of CI was estimated as the rate of rotenone-sensitive NADH oxidation. At the end of the rotenone-insensitive activity recording, a freshly prepared mixture containing phosphoenolpyruvate (PEP), ATP, MgCl_2_, and lactate dehydrogenase (LDH) + pyruvate kinase (PK) was added to the cuvette. This mixture triggers the ATP hydrolase activity of complex V, which can be inhibited by oligomycin as long as the F0 and F1 components of the enzyme remain bound together. The composition of the mixture in each cuvette was: 5 μL of ATP, 50 mM; 5 μL of PEP, 200 mM; 5 μL of MgCl_2_, 500 mM; and 20 μL of PK + LDH. After 1 min, 3 μL of oligomycin (2.5 mM) was added. Under this condition, maximal complex V activity and maximal oligomycin effect were measured after 1 min. Results were then converted into specific activities using the mean of the three values obtained for each sample. CI and CV activities are expressed as nanomoles of oxidized NADH/min/mg protein, using the extinction coefficient ε = 6.22 mM^−1^cm^−1^.

In the second assay, the activity of complex III (CIII: coenzyme Q-cytochrome C reductase) was measured by adding reduced decylubiquinone and a solution of cytochrome *c* (CytC). Next, the formation of reduced cytochrome *c* was followed as previously described.^102^ At the beginning, each cuvette contained 900 μL of sucrose buffer (250 mM sucrose, 50 mM Tris, 1 mM EDTA; pH 7.2), 50 μL of CytC (1 mM), 10 μL of KCN (200 mM), and 30 μL of homogenate; experiments were performed in triplicate. Each cuvette was kept 10 min at 30°C and the absorbance was monitored at 550 nm for 2 min to determine the rate of background CytC reduction. A solution of 10 mM of decylubiquinone was reduced using a crystal of potassium borohydride in 200 μL to produce decylubiquinol. To neutralize the samples, 5 μL of 100 mM HCl with gentle mixing was added until the yellow solution became colorless. Then, 5 μL of colorless decylubiquinol was added to each cuvette and the absorbance was monitored during 2 min. The incubation was pursued by adding 4 μL of antimycin A (200 μM) to determine the rate of antimycin-insensitive activity. The degree of CytC reduction was calculated using the extinction coefficient of 27.2 mM^−1^ cm^−1^. Results were next converted to specific activities using the mean of the three values obtained and the protein concentration estimated by the Bradford method. The activity of CIII was expressed as nanomoles of reduced CytC/min/mg protein.

In the third assay, the activity of complex IV (CIV: cytochrome *c* oxidase [ferrocytochrome c: oxygen oxidoreductase]) was measured by adding a reduced cytochrome *c* (CytC) solution of 2 mM in distilled water. The CytC was reduced by adding a few grains of sodium dithionite; the solution changed color from brown to orange-pink when the reduction was complete.

The test was performed in triplicate at 37°C; each reaction cuvette contained the appropriate buffer: 10 mM of KH_2_PO_4_, pH 7.2, and BSA (1 mg/mL) and the reduced CytC. The reaction was initiated by adding 5 μL of the homogenate. The absorbance changes were monitored at 550 nm for 2 min. The rate of CytC oxidation was calculated using the extinction coefficient of 27.2 mM^−1^ cm^−1^. The activity of CIV was expressed as nanomoles of oxidized CytC/min/mg protein.

Next, the enzymatic activity of two proteins of the TCA cycle, i.e., citrate synthase (CS) and malate dehydrogenase (MDH), were assessed following respectively previous published protocols.^102^^,^^103^

The activity of CS was measured, as an accurate indicator of functional mitochondrial content; it was obtained by the difference of absorbance between DTNB [Ellman reagent, 5,5-dithio-bis-(2-nitobenzoic-acid), Sigma] and its reduced form in the presence of thiol groups. The thiol group (SH) of Coenzyme A reacts with DTNB (colorless) to form 5-mercapto-2-nitrobenzoic acid (TNB^2^), absorbance peak at 412 nm (yellow). Thus, the appearance of TNB^2^ was quantified at 37°C by the measure of absorbance at 412 nm. Each test was performed in triplicate, in 1 mL of 10 mM of KH_2_PO_4_, pH 7.8, 5 μL of Triton 10%, 5 μL of 20 mM Acetyl-CoA 20, and 1 μL of 100 mM DNTB. Next, 5 μL of cerebellar homogenate was tested, the absorbance changes at 412 nm were monitored for 2 min and 5 μL of 10 mM oxaloacetate was added and the absorbance monitored for 2 min again. Results were next converted into specific activities using the mean of the three values obtained for each sample. The CS activity was expressed as nanomoles of TNB^2^/min/mg protein, using the extinction coefficient ε = 13.7 mM^−1^cm^−1^.

The activity of MDH was measured as described.^103^ The reaction was performed in 1 mL of 100 mM of KH_2_PO_4_ pH 7.5 containing 0.14 mM NADH and 30 μL of 7.6 mM oxaloacetate. Five microliters of homogenate was added to each cuvette and absorbance changes were monitored at 340 nm during 2 min at 25°C; each sample was made in triplicate. Results were next converted into specific activity using the mean of the three values obtained for each sample. The MDH activity was expressed as nanomoles of oxidized NADH/min/mg protein, using the extinction coefficient ε = 6.22 mM^−1^cm^−1^.

Protein concentration was quantified by the Bradford assay, following the supplier’s instructions; the measurements were performed in triplicate, using 3 μL of homogenate per sample. The mean values calculated from the triplicates of each assay were used to estimate the specific activities for each complex.

All the chemicals used for the assays were of the highest grade available from Sigma-Aldrich.

### UHPLC-MS/MS analyses

The system Acquity UPLC-Xevo TQD from Waters (Milford, MA, USA) was used for determining the contents of adenosine 5′-triphosphate (ATP), adenosine 5′-diphosphate (ADP), and adenosine 5′-monophosphate (AMP).^56^

The determination of nucleotide contents was performed in frozen cerebellar tissues (20–60 mg/mL) homogenized in a cold mixture of water and methanol (1/3) at 4°C. The samples were centrifuged for 20 min at 15,000 rpm at 4°C. The obtained supernatants were then analyzed by UHPLC-MS/MS. Chromatographic separation was carried out in a gradient using an Acquity UPLC Kinetex C8 column (2.1 mm × 100 mm, 1.7 μm, Phenomenex) as stationary phase, at 35°C. As a mobile phase, a 20-mmol/L binary gradient of ammonium acetate in water (Phase A) and methanol: water (80:20) (Phase B) was used. The gradient was as follows: 0–2 min, 30% B; 2–6 min, 30%–80% B; 6–6.1 min, 80% B; 6.1–10 min, 30% B. The maximum analytical signal was obtained using the following conditions: capillary voltage of 3.5 kV, desolvation temperature at 400°C, the nitrogen and desolvation flow were 301 and 850 L/h, respectively. Multiple reaction monitoring (MRM) was performed to quantify the different analytes. For each compound the optimized parameters were both the quantification and confirmation sonic ion precursor transitions, the cone energy (V) and the energy charge (EC) (eV). The data were processed using the MassLynx 4.1 software (Waters Corporation, Manchester, UK). The result was expressed in nmol/mg of proteins.^56^ In addition, the energy charge (EC) in cerebellar samples was calculated as a regulatory parameter of metabolism^51^ using the formula: EC = ([ATP] + 0.5 [ADP])/([ATP] + [ADP] + [AMP].

### Biomarkers of oxidative stress

The simultaneous determination of the reduced/oxidized aminothiols GSH/GSSG, cysteine/cystine, and homocysteine/homocystine was performed as follows: frozen cerebellar samples were homogenized in PBS with 10 mM N-ethylmaleimide (NEM). Perchloric acid was added to obtain a final concentration of 4%; then, samples were centrifuged at 15,000 × *g* for 15 min at 4°C. The concentration of analytes was determined in the supernatants by UHPLC-MS/MS. The method used has been previously decribed for blood samples from piglets by Escobar and colleagues.^55^

### Statistical analysis

Statistical analyses were performed with GraphPad Prism software version 10.2 for Windows (San Diego, CA, USA, www.graphpad.com). Since we compared more than two unmatched groups, we need to know whether the groups follow a Gaussian (normal) distribution before performing statistical evaluations. Hence, the following normality tests were applied: D’Agostino-Pearson Anderson-Darling test, Kolmogorov-Smirnov, and Shapiro-Wilk. In the case of parametric data, two-way ANOVA (analysis of variance) and the Tukey’s post hoc test for multiple comparisons was used allowing comparison of every mean with every other mean of each group. Some data, specified in Table S3, we also subjected to the Dunnett test to compare every mean with a control mean that yields narrower confidence intervals. The two-way ANOVA was used since our collected data on either control or *Hq* mice treated with three different vectors were compared with independent variables such as number of neurons, transduction yield, behavior, etc.

For nonparametric data, Kruskal-Wallis, also known as one-way ANOVA on ranks, followed by Dunn’s multiple comparisons test was performed; indeed, this test does not assume a normal distribution of the groups.

The significance level was set at α = 0.05. The adjusted *p* values calculated by the software and illustrated in each graphical representation are as follows: ns, *p* > 0.05; ∗*p* ≤ 0.05; ∗∗*p* ≤ 0.01; ∗∗∗*p* ≤ 0.001; ∗∗∗∗*p* ≤ 0.0001. The detailed statistical analyses of each figure are available in Table S3.

For statistical calculations of TEM images, two types of tests were carried out: (1) one-sided Fisher’s exact test for the morphology of mitochondria inside Purkinje cell somas, and (2) the Wilcoxon-Mann-Whitney for the synaptic densities on the somas of neurons in the deep nuclei. To finish the analyses, all the *p* values were corrected by Holm’s method.

## Data and code availability

The data that support the findings of this study are available from the corresponding author, upon reasonable request.

## Supplemental information

Document S1. Figures S1–S3 and Tables S1–S3

Document S2. Article plus supplemental information
