# Supplementary material for: Neuroglobin overexpression in cerebellar neurons of Harlequin mice improves mitochondrial homeostasis and reduces ataxic behavior
Source: Mol Ther. 2024 May 24;32(7):2150–75. doi: 10.1016/j.ymthe.2024.05.030 (PMC11286817; doi:10.1016/j.ymthe.2024.05.030)
Supplement: Document S1. Figures S1–S3 and Tables S1–S3 [file mmc1.pdf]

YMTHE, Volume *32*

Supplemental Information

Neuroglobin overexpression in cerebellar neurons of *Harlequin* mice improves mitochondrial homeostasis and reduces ataxic behavior

Hélène Cwerman-Thibault, Vassilissa Malko-Baverel, Gwendoline Le Guilloux, Edward Ratcliffe, Djmila Mouri, Isabel Torres-Cuevas, Ivan Millán, Bruno Saubaméa, Virginie Mignon, Odile Boespflug-Tanguy, Pierre Gressens, and Marisol Corral-Debrinski

Figure S1. Vector Maps

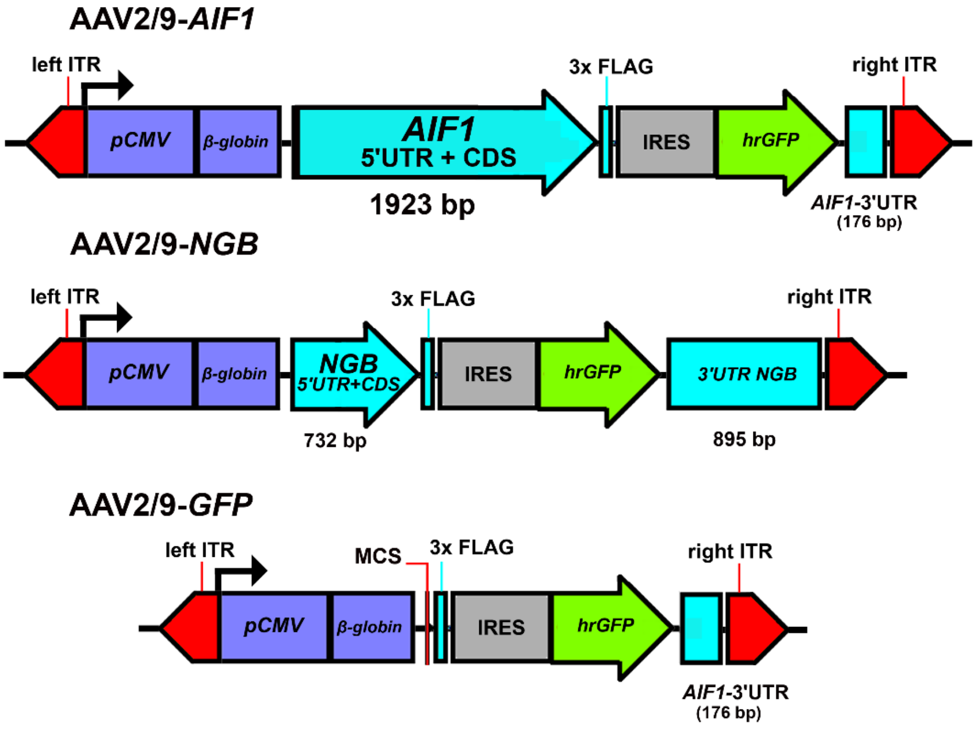

Figure S1. Vector Maps

The three vectors which were administered into cerebellar hemispheres of control and *Hq* mice are shown as diagrams. Original plasmids were obtained from Agilent, the sequences were synthesized by GenScript as well as the cloning in the pAAV-IRES-*hrGFP* vector. Next, vectors were produced by the Translational Vector Core of the INSERM UMR1089 research unit (Nantes, France). The AAV2/2-*Aifm1* vector contains the 5′ UTR (87 bp), the entire ORF (1836 bp), and the 176-bp full-length 3′ UTR of the mouse *Aif* mRNA (NM_012019). The AAV2/2-*Ngb* vector contains the open reading frame (ORF) (453 bp), the 5′ untranslated region (UTR) (279 bp), and the 3′ UTR (895 bp) of the mouse *Ngb* mRNA (NM_022414.2). Both ORFs are transcribed under the control of the cytomegalovirus promoter (pCMV) and the β-globin intron. The plasmid contains a cassette that allows expression of the recombinant humanized green fluorescent protein (GFP) translated from the encephalomyocarditis virus internal ribosome entry site (IRES). As negative control the pAAV-IRES-*hrGFP* vector, which did not include any transgene but possesses the *Aifm1* 3'UTR signal is also illustrated.

<u>Figure S2:</u> Subcellular localization of different mitochondrial proteins in Purkinje cells

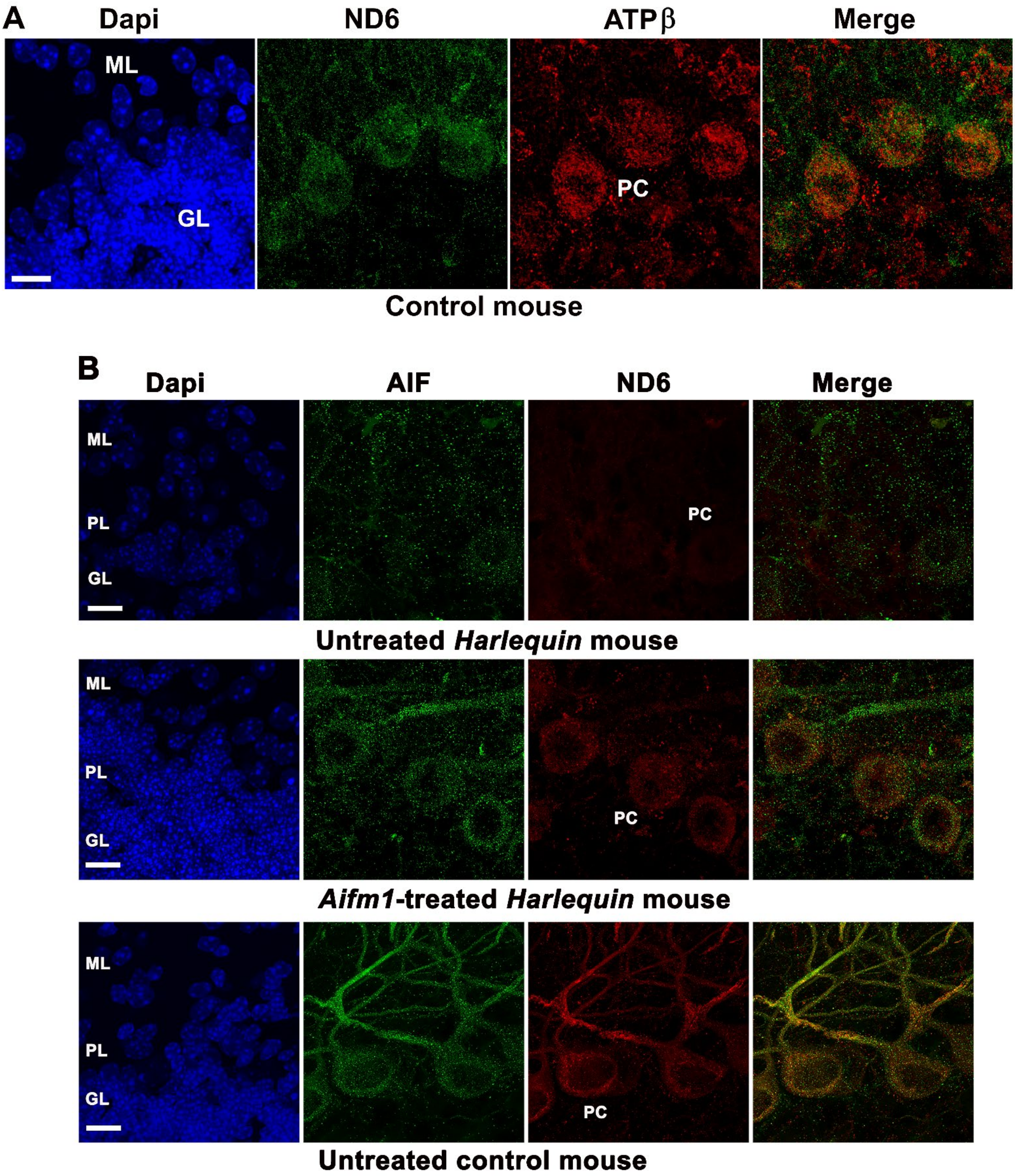

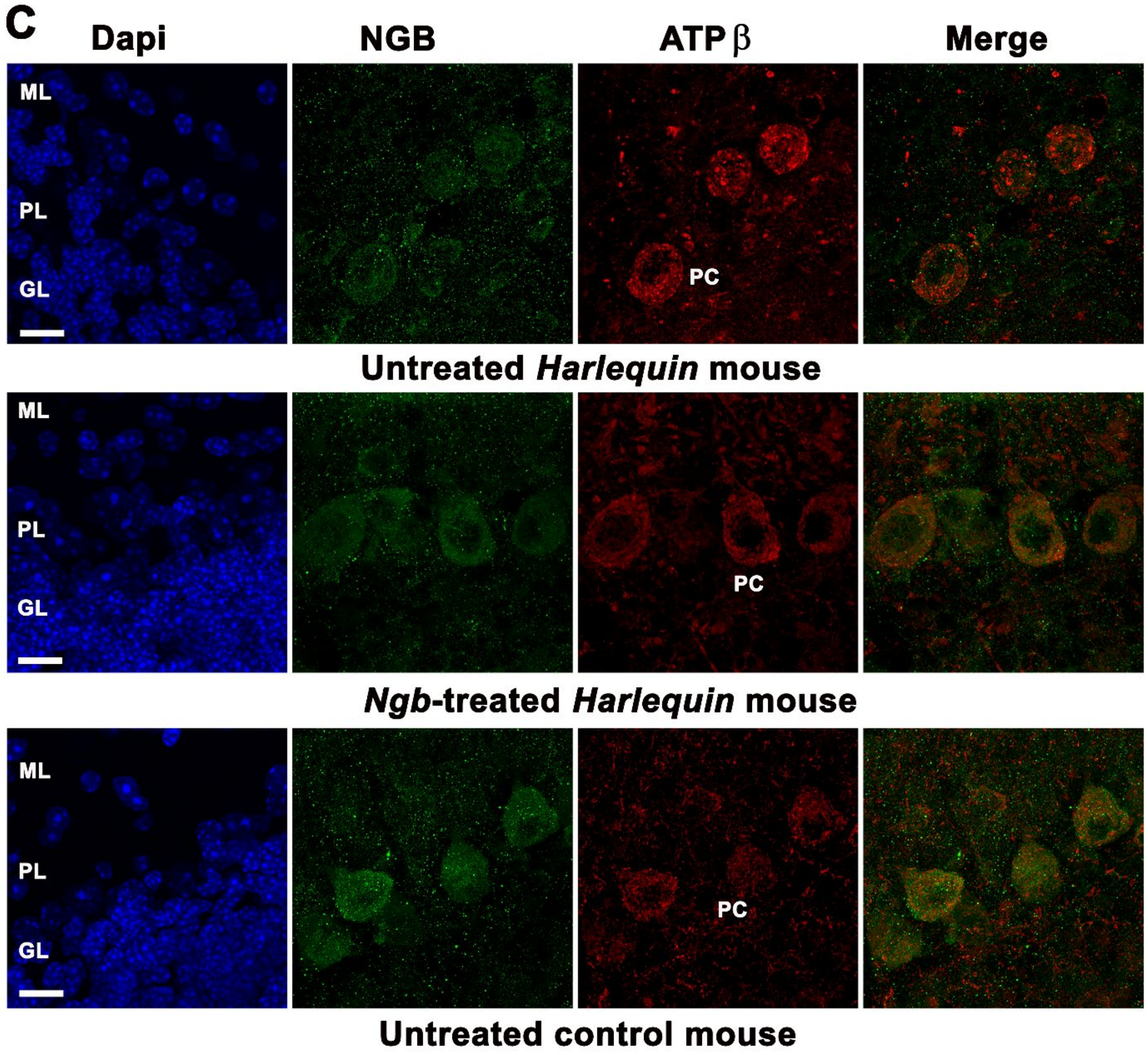

<u>Figure S2:</u> Subcellular localization of different mitochondrial proteins in Purkinje cells

A. Immunohistochemistry was performed in cerebellar sections from untreated control mice aged 8 months, using the combination of antibodies against ATP synthase subunit β (a component of Complex V) and ND6 (a subunit of Complex I).

B. Immunohistochemistry was performed in cerebellar sections from untreated control mice and untreated *Harlequin* mice or *Aifm1*-treated *Harlequin* mice using antibodies against ND6 and AIF.

C. Immunohistochemistry was performed in cerebellar sections from untreated control mice, untreated *Harlequin* mice or *Ngb*-treated *Harlequin* mice using antibodies against NGB and ATP β. Confocal images illustrated correspond to a magnification with a 63X objective applying a 2.5 zoom (scale bar is 25 µm). Images shown correspond to nuclei labeled with Dapi (blue) as well as a composite image (Merge) of the staining obtained with the antibodies: green (AIF or NGB) and red (ND6 or ATP β). Abbreviations: ML, molecular layer; GL, granular layer; PL, Purkinje cell layer; PC, Purkinje Cells. The amounts of primary and secondary antibodies used are shown in Supplemental Table 1.

Figure S3: Gene therapy and features of Purkinje cell dendritic arborizations

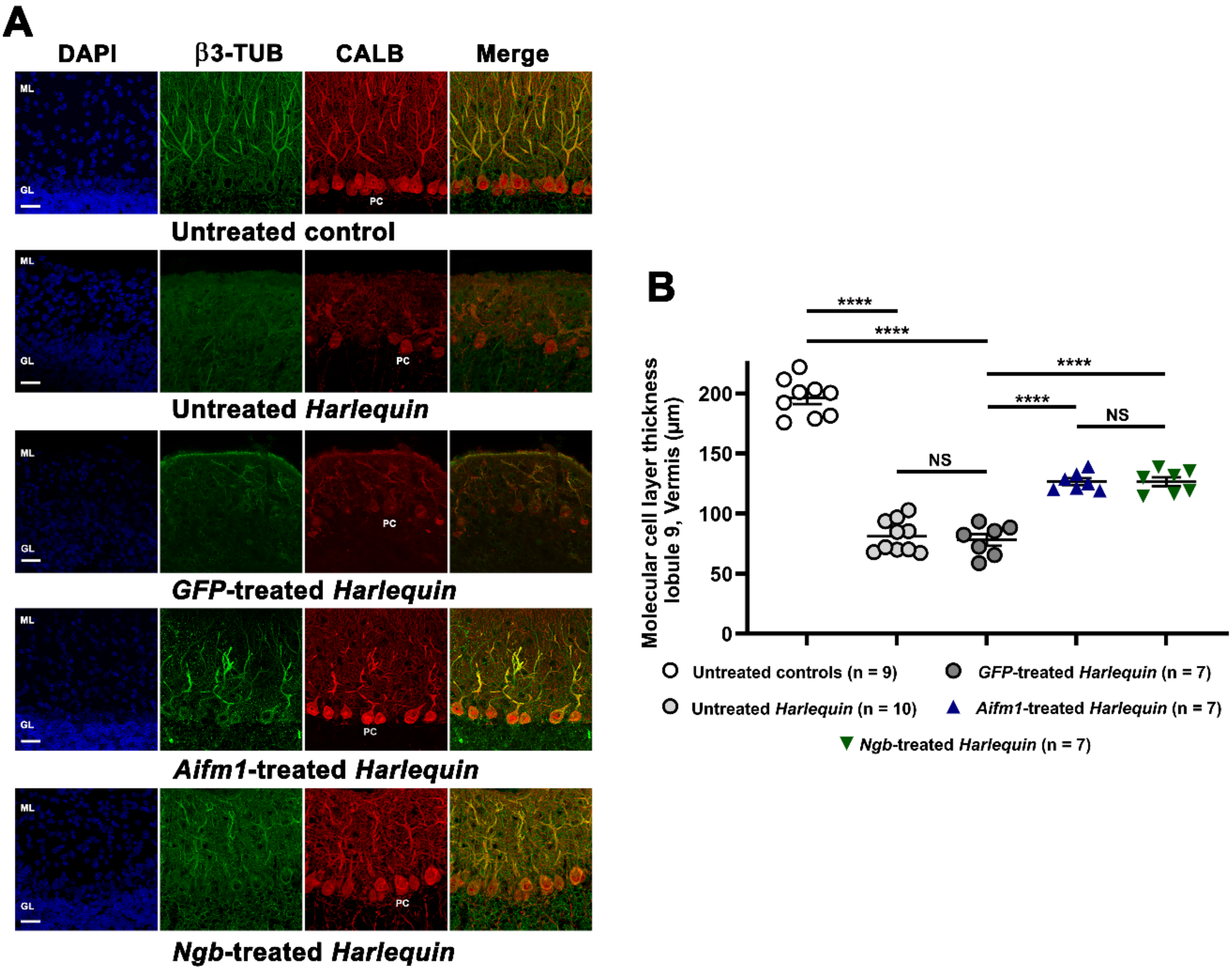

Figure S3: Gene therapy and features of Purkinje cell dendritic arborizations

A. Confocal images from cerebellar sections labeled with Dapi (blue), β3-TUB (green) and Calbindin, CALB (red); are illustrated from an untreated control mouse, an untreated *Hq* mouse, one *Aifm1*-treated *Hq* mouse and one *Ngb*-treated mouse. Dendritic arborizations of Purkinje cells are better preserved in *Aifm1*- and *Ngb*-treated *Hq* sections than in age-matched untreated *Hq* mice. Images were obtained with a Leica TCS SP8 confocal microscope and the LAS X software; scale bars correspond to 25 µm (63X objective).

Abbreviations: ML, molecular layer; GL, Granular cell layer; PC, Purkinje Cells.

B. The thickness of the molecular layer, as an estimate of the total dendritic length, was determined in 6 different points of the lobule IX in reconstructed cerebellar sections from the vermis labeled with CALBINIDIN and β3-TUBULIN antibodies. In the histogram means of the molecular layer thickness in µm ± S.E.M are represented and they correspond to measurements obtained for up to eight independent slices per untreated or treated mice. The numbers of mice evaluated for each condition are indicated in the legend corresponding to each bar. *P* value calculations were obtained with the Graph Pad Prism 10.2 program, *P* values illustrated in histograms are as follow: $P > 0.05$: NS (not significant); $P \leq 0.05$: *; $P \leq 0.01$: **; $P \leq 0.001$: ***; $P \leq 0.0001$: ****. The detailed statistical analyses for the data are available in Supplemental Table 3.

Table S1. Antibody Description

Abbreviations: IIF, indirect immunofluorescence

* The human (Q9NPG2) and mouse (Q9ER97) sequences for the neuroglobin protein have 94% identity (151 amino acids). Concerning the two peptides chosen for raising antibodies in rabbits, they are identical except for amino acid # 46: the cysteine in the human protein is replaced by a glycine in the mouse protein.

Antibody target or reagent	Type	Assay: concentration	Supplier, Catalog no.
Calbindin D-28k	Monoclonal	IIF:1µg/mL	Swant, CB300
Calbindin D-28k	Polyclonal	IIF: 0.5 µg/mL	Merck-Millipore, ABN2192
Neuroglobin	Polyclonal	IIF: 4 µg/mL	Sigma-Aldrich, N-7162
Neuroglobin	Polyclonal	Western: 2 µg/mL	Eurogentec: 2 immunogen peptides against the human protein; amino acids 43–58 and 136–151*
Neuroglobin	Polyclonal	Western: 1 µg/mL	Cliniscience, LSBio LS-C334745
β3-Tubulin	Polyclonal	IIF: 1 µg/mL	Abcam, Ab18207
β-actin	Monoclonal	Western: 0.4 µg/mL	Sigma-Aldrich, clone AC-74, A5316
Tomm20	Monoclonal	Western: 1 µg/mL	Abcam, Ab56783
AIF	Polyclonal	IIF: 2.5 µg/mL<br>Western: 0.05 µg/mL	Abcam, Ab32516<br>[E20], mitocondrial marker
AIF	Monoclonal	IIF: 2.5 µg/mL<br>Western: 0.05 µg/mL	ThermoFisher Scientific, MA5-15880<br>Clone 4E7E11
ND6	Polyclonal	IIF: 1 µg/mL	ThermoFisher Scientific, PA5- 103954
ATP synthase subunit β	Monoclonal	IIF: 1 µg/mL	Thermo-Fischer Scientific, A-21351 (Clone 3D5AB1)
NDUFA9	Monoclonal	IIF: 1 µg/mL	Thermo-Fischer Scientific,459100 (Clone 20C11B11B11)
Alexa 488	Anti-IgG, rabbit	IIF: 4 µg/mL	Life Technologies, A11008
Alexa 594	Anti-IgG, mouse	IIF: 4 µg/mL	Life Technologies, A11005
Goat anti-rabbit IgG	Goat anti-rabbit IgG, horseradish peroxidase conjugate	Western: 0.05 µg/mL	Jackson ImmunoResearch Laboratories, 111-035-144
Goat anti-mouse IgG	Goat anti-mouse IgG, horseradish peroxidase conjugate	Western: 0.05 µg/mL	Jackson ImmunoResearch Laboratories, 115-035-003
DAPI (4′,6-diamidino-2-phenylindole, dihydrochloride)	Nucleic acid stain	IIF: 2 µg/mL	Life Technologies, D1306

<u>Table S2</u>. Primer Pairs used in the qPCR Assays

Transcript	Forward, 5′–3′	Reverse, 5′–3′
Neuroglobin	ATGCTGCAGTGACCAACGTG	GCGGTCCTTGTAGCTGGTGT
Nd4	TCTGCTTACGCCAAACAGATT	GTGATGATGTGAGGCCATGT
Aifm1	CGAGCCCGTGGTATTCGA	CCATTGCTGGAACAAGTTGC
Calbindin	CAGGGAATCAAAATGTGTGGGA	CACACAGATCTTTCAGCAAAGCA
Rpl13a	GCGGATGAATACCAACCCCT	GAGGGATCCCATCCAACACC

Table S3. Statistical Analyses

The overall statistical analyses for each test were performed with the GraphPad Prism 10.2 program. The significance level was set at $\alpha = 0.05$.

N/A: not applicable.

The Gaussian distribution of groups was determined as recommended by the GraphPad Prim software. In the case of parametric data, two-way ANOVA (analysis of variance) followed by Tukey's post hoc test for multiple comparisons was used; this test compares three or more sets of measurements (every mean with every other mean). For some data, we also used the Dunnett test which compares every mean to a control mean. Thus, the test becomes more powerful to detect differences by assuming that all the data are sampled from populations with the same standard deviation, even if the means are different.

For nonparametric data, Kruskal-Wallis, also known as one-way ANOVA on ranks, followed by Dunn's multiple comparisons test was performed; indeed, the Kruskal-Wallis test does not assume a normal distribution of the groups.

Figures	Normality tests	Two-Way Anova (Multiple comparison test)	Kruskal-Wallis (One-Way Anova on ranks)
*Figure 1A: B*ody weights of mice subjected to gene therapy	Anderson-Darling, D'Agostino & Pearson, Shapiro-Wilk and Kolmogorov-Smirnov	Tukey	N/A
Figure 1B: Ratios of cerebellar weight to body weight of mice subjected to gene therapy	Anderson-Darling, D'Agostino & Pearson	Tukey and Dunnett's	N/A
Figure 2C: Transduction yield of Purkinje cells in control and *Harlequin* mice	Shapiro-Wilk	Tukey	N/A
Figure 3C: Purkinje cell numbers	None	N/A	Dunn
Figure 3D: Purkinje cell layer length	Shapiro-Wilk and Kolmogorov-Smirnov	Tukey	N/A
Figure 3D: Purkinje cell number per mm	Shapiro-Wilk and Kolmogorov-Smirnov	Tukey and Dunnett's	N/A
Figure 4A-D: Abundance of several mitochondrial proteins in the cerebella from *Harlequin* mice (qPCRs)	*Aifm1* mRNA: Kolmogorov-Smirnov, *ND4* mRNA: Shapiro-Wilk and Kolmogorov-Smirnov *Ngb* and *Calb* mRNAs did not follow a Gaussian distribution	Tukey (*Aifm1* and *Nd4* mRNAs)	Dunn (*Ngb* and *Calb* mRNAs)
Figure 4F: Abundance of several mitochondrial proteins in the cerebella from *Harlequin* mice (Western blots)	AIF / β-actin: Shapiro-Wilk and Kolmogorov-Smirnov, NDUFA9 / β-actin and TOMM 20 / b-actin: Shapiro-Wilk; NGB / β-Actin and ATP-β / β-actin: Shapiro-Wilk and Kolmogorov-Smirnov	Tukey and Dunnett's (TOMM 20 / b-actin)	N/A
Figure 5: Morphological studies (Intensity of Fluorescence for AIF (C)	Shapiro-Wilk and Kolmogorov-Smirnov	Tukey	N/A
Figure 5: Morphological studies Intensity of Fluorescence for NGB (D)	Shapiro-Wilk and Kolmogorov-Smirnov	Tukey	N/A
Figure 7A: Energetic metabolism (Complex I)	D'Agostino & and Shapiro-Wilk	Tukey and Dunnett	N/A

Figure 7A: Energetic metabolism (Complex III)	D'Agostino & Pearson, Anderson-Darling, Shapiro-Wilk and Kolmogorov-Smirnov	Tukey and Dunnett	N/A
Figure 7A: Energetic metabolism (Complex IV)	Shapiro-Wilk	Tukey and Dunnett	N/A
Figure 7A: Energetic metabolism (Complex V)	None	N/A	Dunn
Figure 7A: Energetic metabolism (Citrate Synthase)	D'Agostino & Pearson, and Shapiro-Wilk	Tukey and Dunnett	N/A
Figure 7A: Energetic metabolism (Malate Dehydrogenase)	D'Agostino & Pearson	Tukey and Dunnett	N/A
Figure 7B: Energetic metabolism (AMP content)	Shapiro-Wilk and Kolmogorov-Smirnov	Tukey and Dunnett	N/A
Figure 7B: Energetic metabolism (ADP content)	None	N/A	Dunn
Figure 7B: Energetic metabolism (ATP content)	None	N/A	Dunn
Figure 7B: Energetic metabolism (Energy Charge)	Shapiro-Wilk	Tukey and Dunnett	N/A
Figure 7B: Oxidative stress (GSH : GSSG)	None	N/A	Dunn
Figure 7B: Oxidative stress (Cysteine : Cystine)	Shapiro-Wilk	Tukey and Dunnett	N/A
Figure 7B: Oxidative stress (Homocysteine : Homocystine)	None	N/A	Dunn
Figure 8A: Behavioral tests (Y-Maze: Time exploring the novel arm)	Anderson-Darling, D'Agostino & Pearson, Shapiro-Wilk and Kolmogorov-Smirnov	Tukey	N/A
Figure 8B: Behavioral tests (Ambulation in the novel arm)	Anderson-Darling, D'Agostino & Pearson, Shapiro-Wilk and Kolmogorov-Smirnov	Tukey	N/A
Figure 8C: Behavioral tests (Open Field arena)	N/A	N/A	Dunn
Figure 8D: Behavioral tests (Whole-limb strength)	Anderson-Darling, D'Agostino & Pearson, Shapiro-Wilk and Kolmogorov-Smirnov	Tukey	N/A
Figure 8E: Behavioral tests (Accelerating Rotarod)	N/A	N/A	Dunn
Figure 8F: Behavioral tests (Open field: Time spent in the center of the arena)	Kolmogorov-Smirnov	Tukey	N/A
Figure 8G: Behavioral tests (Tail Suspension Test)	D'Agostino & Pearson Shapiro-Wilk, Anderson-Darling and Kolmogorov-Smirnov	Tukey	N/A
